# Mapping the landscape of genetic dependencies in chordoma

**DOI:** 10.1038/s41467-023-37593-8

**Published:** 2023-04-06

**Authors:** Tanaz Sharifnia, Mathias J. Wawer, Amy Goodale, Yenarae Lee, Mariya Kazachkova, Joshua M. Dempster, Sandrine Muller, Joan Levy, Daniel M. Freed, Josh Sommer, Jérémie Kalfon, Francisca Vazquez, William C. Hahn, David E. Root, Paul A. Clemons, Stuart L. Schreiber

**Affiliations:** 1 Broad Institute of Harvard and MIT, Cambridge, MA 02142 USA; 2 Chordoma Foundation, Durham, NC 27702 USA; 3 Dana-Farber Cancer Institute, Boston, MA 02215 USA; 4 Harvard University, Cambridge, MA 02138 USA; 5 Present Address: Kojin Therapeutics, Boston, MA 02210 USA; 6 Present Address: University of California San Diego, La Jolla, CA 92093 USA; 7 Present Address: Melanoma Research Alliance, Washington, D.C. 20005 USA

**Keywords:** Cancer genetics, Bone cancer, Functional genomics

## Abstract

Identifying the spectrum of genes required for cancer cell survival can reveal essential cancer circuitry and therapeutic targets, but such a map remains incomplete for many cancer types. We apply genome-scale CRISPR-Cas9 loss-of-function screens to map the landscape of selectively essential genes in chordoma, a bone cancer with few validated targets. This approach confirms a known chordoma dependency, *TBXT* (*T*; brachyury), and identifies a range of additional dependencies, including *PTPN11, ADAR, PRKRA, LUC7L2, SRRM2*, *SLC2A1, SLC7A5, FANCM*, and *THAP1. CDK6, SOX9, and EGFR*, genes previously implicated in chordoma biology, are also recovered. We find genomic and transcriptomic features that predict specific dependencies, including interferon-stimulated gene expression, which correlates with *ADAR* dependence and is elevated in chordoma. Validating the therapeutic relevance of dependencies, small-molecule inhibitors of SHP2, encoded by *PTPN11*, have potent preclinical efficacy against chordoma. Our results generate an emerging map of chordoma dependencies to enable biological and therapeutic hypotheses.

## Abstract (web summary)

Cancer cells possess unique molecular features that can confer an increased dependence on specific genes. Here, the authors use CRISPR-Cas9 screens to identify selectively essential genes and therapeutic targets in chordoma.

## Introduction

Elucidating the set of genes upon which cancer cell proliferation and survival are especially reliant (the “dependencies” of cancer) can provide an important roadmap for the treatment and characterization of cancer. Knowledge of these vulnerabilities can guide the choice of cancer-selective drugs, reveal mechanisms of cancer cell initiation or maintenance, and permit the classification of cancer subtypes or cell states^1^. The observation that clinically relevant dependencies can be predicted by somatic mutation or copy-number alteration of the same gene (exemplifying the phenomenon of “oncogene addiction”) has motivated large-scale efforts to sequence tumor genomes and thereby facilitate cancer-dependency discovery^2,3^. However, identifying tractable therapeutic vulnerabilities using tumor genome sequencing has been more challenging for cancer types that harbor relatively few somatic mutations, when gene mutations that enhance fitness cannot be readily distinguished from bystander mutations, or when altered genes cannot be modulated with the existing arsenal of drugs.

Alongside efforts to identify somatically mutated cancer-dependency genes, a growing number of tumor-intrinsic cancer dependencies have been discovered via functional approaches whose classification extends beyond that of canonical mutated oncogenes^1,4^. These include dependencies reflecting synthetic lethal interactions^5,6^, such as those occurring when the loss of one member of a paralog pair leads to increased reliance on the other member (“paralog dependencies”)^7,8^, or those resulting from hemizygous copy-number loss and/or reduced expression of the same gene (“CYCLOPS” genes)^9^; dependencies arising from dysregulated or persistent expression of master regulatory genes that mediate normal lineage development (“lineage-survival” dependencies)^10^; and dependencies unique to a particular cancer cell state^11^. In this way, many cancer dependencies can be classified as “non-oncogene” dependencies, whereby the unique molecular features of the cancer cell confer a heightened dependence on the normal cellular function of specific genes^12^.

Indeed, dependencies resulting from somatically mutated oncogenes now appear to represent only a minority of cancer dependencies overall^4^. Notably, cancer types that harbor relatively few somatic mutations, such as some pediatric cancers, do not necessarily have a reduced number of genetic dependencies relative to cancer types with a higher mutational burden^13^. Together, these observations suggest the existence of a spectrum of cancer dependencies that may not be discoverable via tumor genome sequencing, but may nevertheless serve as effective therapeutic targets. Yet, for many cancer types, a comprehensive map of differential dependencies from which such targets could be nominated remains incomplete.

One such cancer type is chordoma, a primary bone cancer for which there is a limited number of validated cancer targets and no approved systemic therapy^14^. Genomic sequencing of chordoma tumors has demonstrated that chordoma is a genomically quiet cancer: recurrent somatic events include amplifications of *TBXT* (encoding brachyury); homozygous deletion of *CDKN2A*; and mutations in PI3K signaling genes, SWI/SNF complex genes, and *LYST*; but nearly half of all sporadic chordoma cases do not harbor any known driver mutation^15,16^. Furthermore, most somatic alterations that do exist are not currently targetable^15,16^—limiting the ability to identify an appropriate targeted therapy in most patients using traditional genome-guided precision medicine approaches. Nonetheless, chordoma tumor cells may have distinct genomic or functional features that render them especially dependent on the activities of specific genes. We sought to identify vulnerabilities that are conferred by the unique cellular circuitry of chordoma, with a view to revealing mechanism-based approaches for the treatment of this disease.

To this end, here we applied genome-scale CRISPR-Cas9 loss-of-function screens to map the landscape of selective dependencies in chordoma. Previously, CRISPR-Cas9 screens in two chordoma cell lines had identified the developmental transcription factor *T* (brachyury), renamed *TBXT*, to be the top selectively essential gene in chordoma, relative to 125 non-chordoma cancer cell lines^17^. While *TBXT* had been the only statistically significant dependency gene identified, we hypothesized that this could be attributed to the small set of chordoma cell lines screened, and that the full range of tumor dependencies in chordoma remained to be discovered. Moreover, *TBXT* encodes a transcription factor that is currently considered challenging to drug, further motivating a search for additional dependency genes.

In this study, by expanding the set of cell lines subjected to genome-scale loss-of-function screening, we recovered *TBXT* and further discovered a spectrum of additional chordoma dependency genes. We demonstrated that targeting one such dependency in preclinical models of chordoma resulted in profound antitumor efficacy, providing a rationale for new clinical trials in chordoma. Together, these findings generate an emerging map of selectively essential genes in chordoma, facilitating future studies that seek to understand the unique tumor biology of this cancer type.

## Results

### Genome-scale CRISPR-Cas9 screens identify a spectrum of selectively essential genes in chordoma

**Fig. 1:**
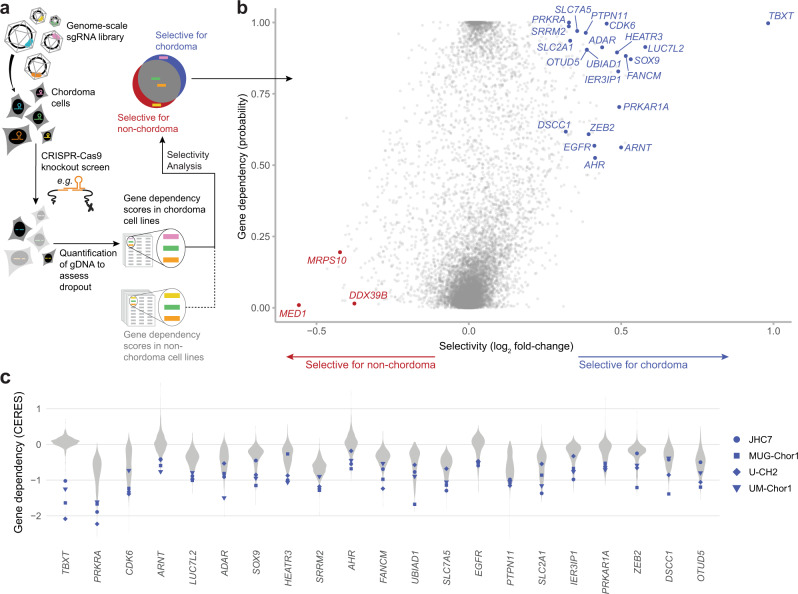
Genome-scale CRISPR-Cas9 screens identify a spectrum of selectively essential genes in chordoma. **a** Experimental workflow of genome-scale CRISPR-Cas9 loss-of-function screens to identify selectively essential genes in chordoma. **b** Selective essentiality analysis identifying chordoma dependency genes. Selectivity is quantified by the log_2_ fold-change in mean dependency probability scores between four chordoma and 765 non-chordoma cell lines (x-axis). The y-axis depicts the median dependency probability score for the chordoma cell lines. Gene dependencies selective for chordoma/non-chordoma are indicated in blue/red (see Methods for details). **c** Distribution of CERES gene effect scores for the indicated genes across four chordoma cell lines (blue) and 765 non-chordoma cell lines (gray; non-chordoma cell lines were profiled as part of the Broad Institute Cancer Dependency Map). Lower CERES gene effect scores indicate higher dependency of a cell line on a given gene. Genes are ranked by decreasing selectivity for chordoma, quantified by the log_2_ fold-change in mean CERES scores between four chordoma and 765 non-chordoma cell lines. Source data are provided as a Source Data file. See also related Supplementary Data 1.

To map the landscape of genes essential for chordoma cell viability, we performed genome-scale pooled CRISPR-Cas9 loss-of-function screens in two chordoma cell lines (JHC7, U-CH2), thereby doubling the number of chordoma cell lines profiled using this approach^17^. The chordoma cell lines used for screening collectively model both sacral and clival chordoma tumors (Supplementary Table 1) and form a distinct cluster by their gene-expression profiles relative to cell lines derived from other tumor types (Supplementary Fig. 1). A library of >74,000 single-guide RNAs (sgRNAs) targeting ~18,560 genes (Methods) was introduced via lentiviral transduction into stably Cas9-expressing chordoma cells. Cells were grown in culture for 20–22 days, after which sgRNAs were quantified from the genomic DNA (gDNA) of surviving cells using massively parallel sequencing. SgRNAs that were depleted from the cell population relative to the screening library were inferred to target candidate essential genes. Gene dependency scores were calculated using two measures: one quantifies the degree of certainty that gene loss impacts cell viability and/or proliferation (dependency probability score, ranging from zero to one), and the other quantifies the magnitude of this impact (CERES gene effect score, with a more negative value indicating higher dependency) (Methods)^18,19^. To identify dependencies selective for chordoma, and remove commonly essential genes, we took advantage of dependency data generated using a large collection of non-chordoma cancer cell lines as part of the Broad Institute Cancer Dependency Map project (DepMap; https://depmap.org/portal/). Notably, the CRISPR-Cas9 screening methodology and sgRNA library we used to profile chordoma cell lines were the same as those used for the DepMap project, permitting consistent data normalization and gene dependency scoring across all cell lines. We thus compared gene dependency scores derived from four chordoma cell lines (JHC7, U-CH2, UM-Chor1, and MUG-Chor1) to those from 765 non-chordoma cancer cell lines that were screened as part of the DepMap project^18^ to identify selectively essential genes in chordoma (Fig. 1a).

Consistent with previous findings, the top selectively essential gene, out of over 18,000 analyzed, was *TBXT* (Fig. 1b and Supplementary Fig. 2)^17^. In addition to *TBXT*, this analysis identified a diverse spectrum of selectively essential genes in chordoma, including *PTPN11*, *ADAR*, *PRKRA*, *LUC7L2*, *SRRM2*, *SLC2A1*, *SLC7A5*, *FANCM*, *AHR*, *ARNT*, *HEATR3*, *UBIAD1*, *IER3IP1*, *PRKAR1A*, *ZEB2*, *DSCC1*, and *OTUD5* (Fig. 1b, c and Supplementary Fig. 2). Genes previously implicated in chordoma biology, including *CDK6*, *SOX9*, and *EGFR*, were also recovered^20–22^. Conversely, a small number of genes was identified whose gene dependency scores were of a greater magnitude in non-chordoma than in chordoma cell lines, and these genes included *MED1*, *MRPS10*, and *DDX39B* (Fig. 1b and Supplementary Fig. 2). We note that while we focused our study on dependencies selective for chordoma, hypothesizing that differential dependencies could be exploited to develop targeted drugs with minimal toxicity, gene dependency scores corresponding to all genes tested in chordoma are available in Supplementary Data 1, 2. In addition, we deposited our screening data on chordoma cell lines in the DepMap repository (10.6084/m9.figshare.12280541.v4) to facilitate additional data exploration and analysis.

**Fig. 2:**
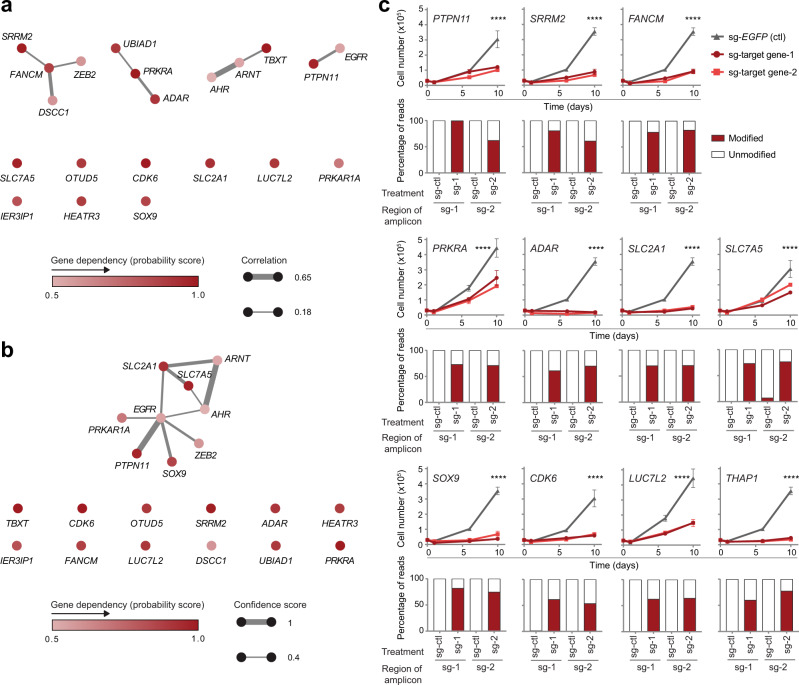
Validation of candidate chordoma dependency genes. **a** Co-essentiality network for selective chordoma dependency genes. Nodes: chordoma dependency genes, colored by dependency probability scores. Edges: Pearson correlation coefficient ≥0.18 between dependency profiles for connected gene pairs, i.e., dependency probability scores across all 769 cancer cell lines; edge width scaled by correlation coefficient. For clarity, we show all genes included in the analysis by listing singletons (genes without connections exceeding our thresholds) below the connected components of the network. **b** STRING protein-protein interaction network for selective chordoma dependency genes. Nodes: chordoma dependency genes, colored by dependency probability scores. Edges: putative interactions with a STRING confidence score ≥0.4; edge width scaled by confidence score. **c** (Top rows) Proliferation of Cas9-expressing UM-Chor1 chordoma cell lines transduced with one of two distinct sgRNAs targeting a candidate dependency gene or a non-targeting sgRNA control. Points represent the mean ± s.d. (*n* = at least 3 biological samples measured in parallel). ****P < 0.0001, derived from a two-way analysis of variance (ANOVA). *P* values for the test comparing sg-*EGFP* and sg-target gene*-*1 are displayed and refer to the time × treatment interaction. Additional details of *P* values and effect sizes are reported in Supplementary Data 7. Graphs with identical sg-*EGFP* control curves reflect experiments performed in parallel on the same day. (Bottom rows) Amplicon sequencing results confirm on-target editing following sgRNA treatment. We quantified the percentage of modified (red) versus unmodified (white) reads of the targeted genomic site following sgRNA treatment of UM-Chor1-Cas9 cells (sg-*EGFP* non-targeting control or one of two distinct sgRNAs targeting a candidate dependency gene). The small fraction of “modified” reads observed for the sg-*SLC7A5*−2-targeted amplicon with sg-*EGFP* control treatment originates from a residual amount of single-nucleotide variation we were unable to match to genomically defined off-target amplicons. However, their distribution points to an additional off-target amplicon as the source (rather than true editing events). Source data are provided as a Source Data file.

To classify and prioritize candidate essential genes in chordoma, we first asked whether these genes could be grouped into shared biological pathways or functions. Functionally related genes often exhibit similar patterns of essentiality across cell lines^23–25^; thus, we measured whether candidate dependency genes display correlated patterns of essentiality across 769 cancer cell lines that had been subjected to CRISPR-Cas9 loss-of-function screening (DepMap, https://depmap.org/portal/; Fig. 2a). Using this approach, four groups of functionally related genes emerged: (1) *FANCM*, *SRRM2*, *ZEB2*, and *DSCC1*; (2) *UBIAD1*, *PRKRA*, and *ADAR*; (3) *AHR*, *ARNT*, and *TBXT*; and (4) *PTPN11* and *EGFR* (Fig. 2a). Most similar were gene pairs encoding proteins with known functional relationships: *AHR* and *ARNT* form a transcriptionally active heterodimer^26^; *FANCM* and *DSCC1* each have roles in various DNA replication-related processes^27,28^; *PTPN11* can act as a positive effector of mitogenic signaling induced by the receptor tyrosine kinase *EGFR*^29^; and *ADAR* and *PRKRA* regulate interferon responses^30^. We note that the correlation between *TBXT* and *ARNT* dependencies is driven by the chordoma cell lines (Supplementary Fig. 3) and thus may reflect the independent co-occurrence of two dependencies in chordoma cells rather than a related biological function of these two genes. Complementing the co-essentiality analysis, we also grouped dependency genes based on publicly available protein-protein interaction annotations (Fig. 2b). This analysis yielded a single cluster of genes comprising *EGFR*, *PRKAR1A*, *PTPN11*, *SOX9*, *ZEB2*, *SLC7A5*, *SLC2A1*, *ARNT*, and *AHR* (Fig. 2b) and recovered known strong connections for *PTPN11*/*EGFR* and *AHR*/*ARNT*. Several candidate genes, including *OTUD5*, *CDK6*, *LUC7L2*, *IER3IP1*, and *HEATR3*, were not linked to other chordoma dependency genes following either of these two approaches (Fig. 2a, b).

We applied this pathway analysis to select a diverse subset of candidate dependency genes for validation and further functional characterization. Genes were selected to represent both those functionally connected to other chordoma dependency genes (*PTPN11*, *FANCM*, *ADAR*, *PRKRA*, *SOX9, SRRM2, SLC2A1,* and *SLC7A5*), as well as those with putatively unique effects (*LUC7L2* and *CDK6*). We included an additional gene for validation, *THAP1*, that is commonly essential across all cancer cell lines, yet whose loss has a greater degree of viability reduction in chordoma cell lines compared to non-chordoma cell lines (Supplementary Fig. 2).

To validate the essentiality of a candidate dependency gene, Cas9-expressing UM-Chor1 chordoma cells were transduced in parallel with two independent sgRNAs targeting a candidate dependency gene and a non-targeting control and assayed for cell viability (Fig. 2c). Consistent with primary screening results, sgRNA-mediated gene suppression of all candidate dependency genes tested led to impaired chordoma cell proliferation (Fig. 2c). SgRNA-mediated gene editing was confirmed for all validated genes via amplicon sequencing of treated cells (Fig. 2c); and sgRNA-mediated protein suppression was measured and confirmed for a subset of validated genes (Supplementary Fig. 4). The high validation rate of the candidate dependency genes tested demonstrates the reliability of the primary screening analysis to identify bona fide chordoma dependency genes, which likely extend beyond those chosen for functional follow-up.

### Genomic and transcriptomic features that predict specific gene dependencies

Identifying a molecular feature that is associated with a specific genetic dependency can help classify tumors by their expected response to targeted therapy and reveal the mechanisms underlying gene essentiality. We thus explored genomic and transcriptomic features that predict specific gene dependencies discovered in our screens. Given the limited power to identify such predictive features using chordoma cell lines alone, we first queried over 700 cancer cell lines profiled as part of the Cancer Cell Line Encyclopedia (CCLE)^31^ for their gene-mutation, gene-copy-number, and gene-expression features. For each chordoma dependency gene, we correlated its signature of essentiality across all cancer cell lines in DepMap with these genomic and transcriptomic profiling data. In addition to calculating correlations, we performed a univariate linear regression for each dependency–feature pair (full results for both analyses are available at 10.6084/m9.figshare.21774746.v1). We then used the resulting predictive features as a discovery set, validating their presence in the chordoma cell lines.

**Fig. 3:**
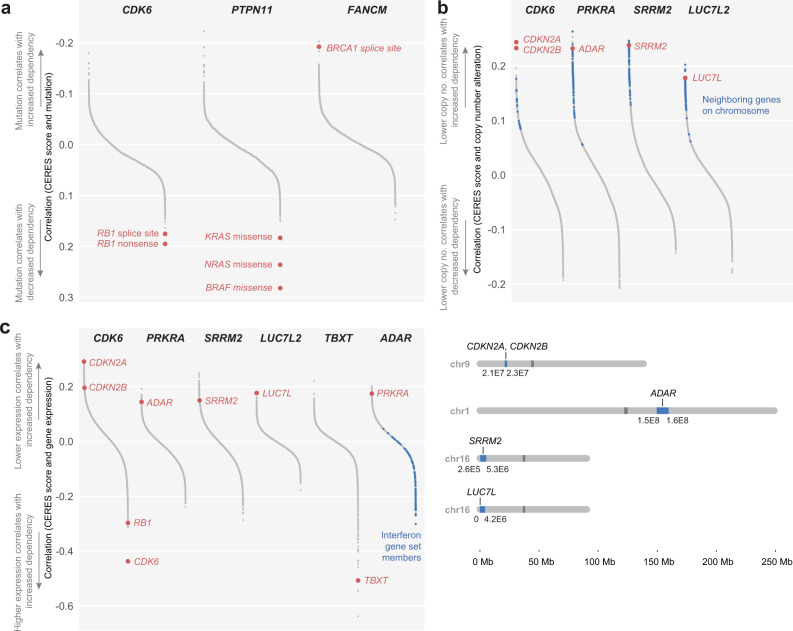
Genomic and transcriptomic predictors of gene dependencies. **a** Mutation correlates of selected dependency genes, ranked by increasing correlation coefficients. The direction of the y-axis was reversed to maintain consistent interpretation across all panels. **b** (Top) Copy-number correlates of selected dependency genes, ranked by decreasing correlation coefficients. Blue: genes neighboring the labeled correlate on the same chromosome, within a window selected for maximum enrichment of neighboring genes at the top of the correlation rank list (see Methods). (Bottom) Genomic loci of copy-number correlates and, in blue, window selected by enrichment analysis. **c** Gene-expression correlates of selected dependency genes, ranked by decreasing correlation coefficients. Members of the MSigDB Interferon Alpha gene set are indicated in blue. For all correlation calculations, we used pairwise-complete observations to handle occasional missing values. All comparisons included at least 719 cell lines, and over 95% of comparisons included the complete set of cell lines for which data were available (mutations: 768; gene expression: 767; copy number: 769). Full results are available at 10.6084/m9.figshare.21774746.v1. See also related Supplementary Data 3.

This analysis recovered mutations in genes known to act in the same pathway as specific dependency genes: decreased dependence on *CDK6* can be predicted by nonsense and splice-site mutations in the downstream target *RB1*^32^; decreased dependence on *PTPN11* can be predicted by missense mutations in the downstream effectors *BRAF*, *NRAS*, or *KRAS*^4,33^; and increased dependence on *FANCM* can be predicted by splice-site mutations in its functional and synthetic lethal partner, *BRCA1*^34^. (Fig. 3a).

We also found copy-number changes in several categories of genes to be associated with specific gene dependencies (Fig. 3b). These included copy-number changes in genes known to act in the same pathway as the dependency gene, such as the previously reported correlation between lower copy number of *CDKN2A*/*CDKN2B* with CDK4/6 dependency^35^; as well as the correlation between *ADAR* copy-number alteration with *PRKRA* dependency. In the case of *SRRM2*, copy-number changes correlate with dependency on the same gene (*SRRM2*). Lastly, this analysis identified copy-number alteration in the genetic paralog of a dependency gene: lower copy number of *LUC7L* is predictive of *LUC7L2* dependency (Fig. 3b).

As expected, correlations between gene dependency and copy-number alterations were similarly reflected at the gene-expression level: dependency on *CDK6*, *PRKRA*, *SRRM2*, and *LUC7L2* each could be predicted by gene-expression changes in *CDKN2A*/*CDKN2B*; *ADAR*; *SRRM2*; and *LUC7L*, respectively (Fig. 3c). *CDK6* dependency could also be predicted by higher gene expression of *RB1* and *CDK6* (Fig. 3c). We also observed high *TBXT* expression to predict dependence on *TBXT*. Notably, this association is driven by chordoma cell lines: only a few non-chordoma cell lines express *TBXT*, and none shows high *TBXT* dependency (Supplementary Fig. 5). This analysis also identified expression of interferon (IFN) genes to be correlated with dependency on the RNA adenosine deaminase *ADAR*, consistent with the observation that high expression levels of interferon-stimulated genes (ISGs) are a biomarker of *ADAR* dependency (Fig. 3c, Supplementary Fig. 6)^36,37^.

Several of the correlated features identified using the set of over 700 cancer cell lines are present in individual chordoma cell lines (Supplementary Data 3) and may have therapeutic relevance. For example, U-CH2 and MUG-Chor1 chordoma cell lines each show biallelic loss of *CDKN2A*, as well as highly selective dependence on *CDK6* (Supplementary Data 3 and Fig. 1c). As somatic homozygous deletion of *CDKN2A* is a recurrent feature of chordoma tumors^15,16,38^, these results suggest that patients whose tumors harbor *CDKN2A* loss may benefit from inhibitors targeting CDK6. In addition to generating therapeutic hypotheses, we also asked whether correlated features could provide insight into the cellular circuitry of chordoma. As ISG expression levels have not been characterized in chordoma, we further investigated this feature in chordoma models.

### Interferon-stimulated genes are overexpressed in chordoma cells and further upregulated following *ADAR* gene suppression

**Fig. 4:**
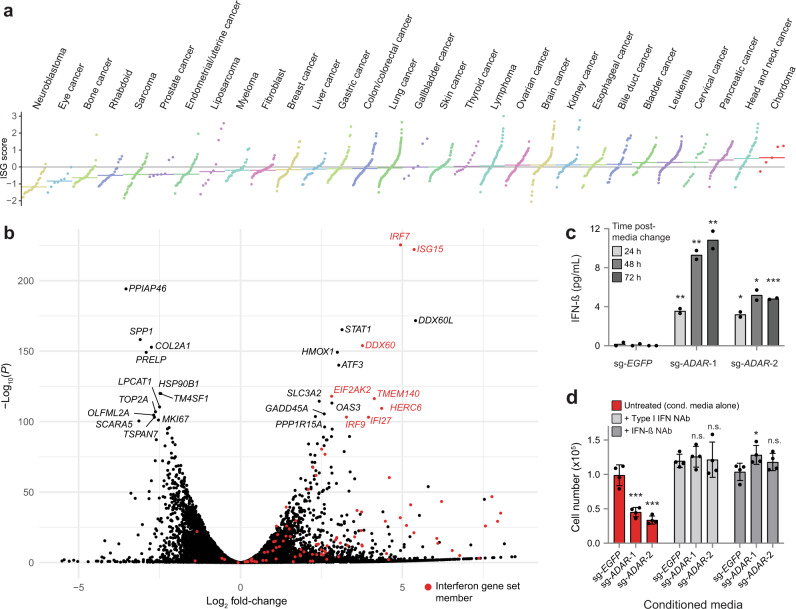
Interferon-stimulated genes are overexpressed in chordoma cells and further upregulated following *ADAR* gene suppression. **a** Distribution of ISG core scores^37^ for chordoma cell lines and 1294 non-chordoma cancer cell lines in the CCLE, grouped by lineage annotation. Colored horizontal bars: median scores for each group. Gray horizontal line: zero-score mark. **b** Differential gene expression comparing the average effects of two distinct *ADAR*-targeting sgRNAs to a non-targeting sgRNA control in Cas9-expressing UM-Chor1 cells. Gene expression was measured with RNA sequencing. *P* values were derived from a Wald test and Benjamini–Hochberg adjusted. Members of the MSigDB Interferon Alpha gene set are indicated in red. **c** IFN-β levels in conditioned media harvested from Cas9-expressing UM-Chor1 cells transduced with the indicated sgRNAs and subsequently subjected to a media change after selection for infected cells. IFN-β levels were measured by ELISA. Data represent the mean of two technical replicates. *P < 0.05, **P < 0.01, ***P < 0.001, derived from a two-tailed, unpaired *t*-test. The statistical test was performed on the indicated condition and the corresponding sg-*EGFP* control. Additional details of *P* values and effect sizes are reported in Supplementary Data 7. **d** Cell viability of parental UM-Chor1 cells treated for 5 days with conditioned media harvested from Cas9-expressing UM-Chor1 cells transduced with the indicated sgRNAs. Treatment with conditioned media was done in the presence or absence of neutralizing antibodies (NAbs) specific to type I IFNs or IFN-β. Data represent the mean ± s.d. (*n* = 4 biological samples measured in parallel). n.s. not significant, *P < 0.05, ***P < 0.001, derived from a two-tailed, unpaired *t-*test. The statistical test was performed on the indicated condition and the corresponding sg-*EGFP* control. Additional details of *P* values and effect sizes are reported in Supplementary Data 7. Source data are provided as a Source Data file. See also related Supplementary Data 5.

Given the association of elevated ISG expression with *ADAR* dependence, and chordoma cells’ selective dependence on *ADAR* relative to non-chordoma cancer cell lines, we compared the expression of ISGs in chordoma cells versus that of other cancer types using a previously described 38-gene signature quantifying IFN pathway engagement (“ISG core score”)^37^. Strikingly, chordoma cell lines have a higher median ISG core score than any of the 29 cancer lineages represented by at least two cell lines in the CCLE (Fig. 4a), suggesting high IFN signaling in chordoma. We confirmed this result using two alternative published IFN gene signatures (Supplementary Fig. 7)^39^, in addition to the 38-gene signature described above.

In other cancer types, *ADAR* suppression in *ADAR*-dependent cells triggers the production of type I IFNs and activation of the nucleic acid sensor PKR, a mediator of IFN-dependent growth arrest^36,37,40^. Consistent with these findings, IFN-alpha- and IFN-gamma-response genes are the most enriched among upregulated genes following sgRNA-mediated repression of *ADAR* in chordoma cells using both gene-set enrichment analysis (GSEA)^41^ (Supplementary Fig. 8a and Supplementary Data 4), as well as a network-based enrichment method (GeLiNEA)^42^ (Supplementary Fig. 8b, c). We observed the upregulation of specific IFNs and ISGs, such as *IRF7* and *ISG15*; as well as *EIF2AK2*, which encodes PKR (Fig. 4b and Supplementary Data 5).

IRF7 is a key regulator of type I IFN gene expression: it activates IFN-β expression and secretion, which in turn stimulates type I IFN receptor to further amplify *IRF7* expression via a positive feedback loop^43,44^. We assayed IFN-β secretion induced by *ADAR* depletion by measuring IFN-β levels in the conditioned media of sg-*ADAR*-treated chordoma cells. Consistent with the observed upregulation of *IRF7* expression, *ADAR* gene suppression induced secretion of IFN-β (Fig. 4c). Furthermore, the conditioned media from *ADAR*-deficient cells was sufficient to reduce proliferation of parental chordoma cells, suggesting that paracrine mechanisms contribute to sg-*ADAR*-induced lethality and can act independently of *ADAR* gene suppression (Fig. 4d). Treatment of conditioned media with neutralizing antibodies specific for either type I IFNs or IFN-β alone rescued this antiproliferative phenotype (Fig. 4d). Consistent with these findings, parental chordoma cells are also sensitive to treatment with recombinant IFN-β (Supplementary Fig. 9). Taken together, these results indicate that chronic ISG expression is elevated in chordoma cells relative to other cancer types, and that *ADAR* gene suppression further upregulates these genes and induces the secretion of factors, such as IFN-β, which can reduce chordoma cell viability in a non-cell autonomous fashion.

### Inhibitors of SHP2, encoded by *PTPN11*, represent candidate therapeutic agents in chordoma

In addition to providing insights into the cellular circuitry of chordoma, identifying a range of chordoma dependency genes may reveal candidate therapeutic targets. Several chordoma dependency genes identified in the primary screens, including *PTPN11*, *EGFR*, and *CDK6*, encode proteins that are currently targetable with small-molecule inhibitors. We investigated the immediate therapeutic relevance of chordoma dependency genes by determining whether small-molecule inhibitors targeting these proteins have antiproliferative activity in chordoma cells. EGFR-inhibitor and CDK4/6-inhibitor sensitivity in chordoma has been described previously^20,21^; thus, we focused on inhibitors of Src homology-2 domain-containing phosphatase 2 (SHP2), the protein encoded by *PTPN11*. SHP2 is a tyrosine phosphatase that promotes signal transduction downstream of receptor tyrosine kinases to activate the RAS/mitogen-activated protein kinase (MAPK) cascade, and has additional functions in modulating tumor immunity^33^; the recent development of selective, allosteric compounds targeting SHP2 led us to investigate their therapeutic potential in chordoma^45–48^.

**Fig. 5:**
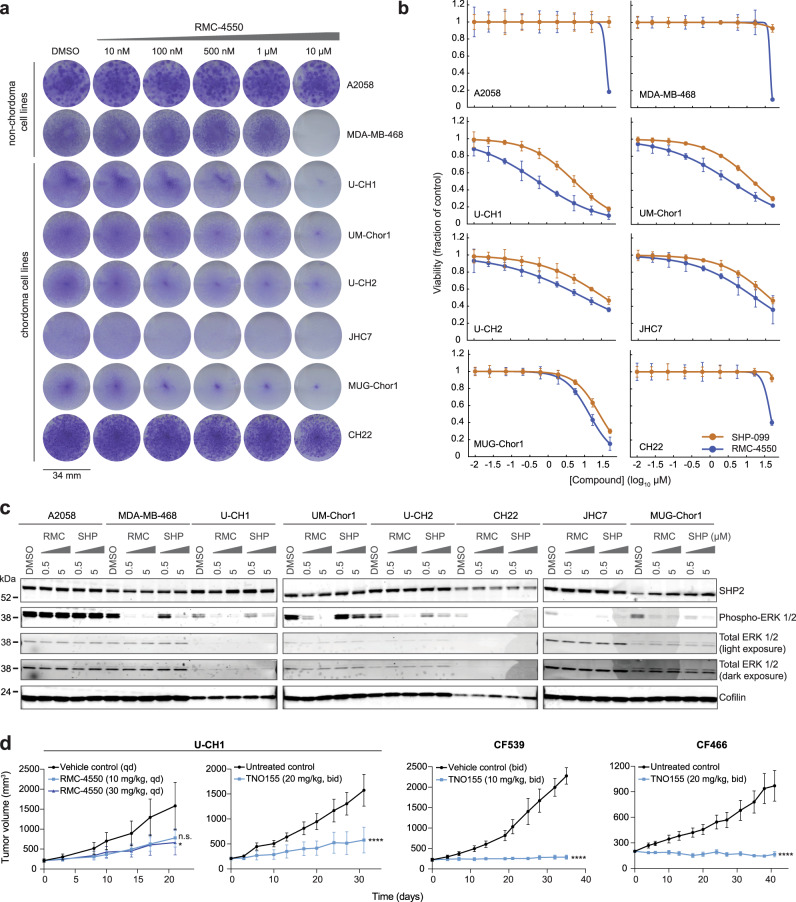
Inhibitors of SHP2, encoded by *PTPN11*, represent candidate therapeutic agents against chordoma. **a** Colony formation assays of chordoma and non-chordoma (negative control A2058; positive control MDA-MB-468) cell lines treated with indicated concentrations of RMC-4550 for 14 days. **b** Viability of chordoma and non-chordoma (negative control A2058; positive control MDA-MB-468) cell lines treated with indicated concentrations of SHP2 inhibitors RMC-4550 and SHP099 and assayed for cell viability after 6 days with CellTiter-Glo. Response data were represented by a fitted curve to the mean fractional viability at each concentration relative to vehicle-treated cells; error bars represent the s.e.m. (*n* = 4 biological samples measured in parallel). **c** Immunoblot analysis of chordoma and non-chordoma (negative control A2058; positive control MDA-MB-468) cell lines treated with indicated concentrations of RMC-4550, SHP099, or DMSO for 2 h. **d** Tumor proliferation in mice engrafted with chordoma cells (U-CH1 cell line-derived xenograft, CF539 PDX, or CF466 PDX) and treated with a SHP2 inhibitor (RMC-4550 or TNO155). Points represent the mean tumor volume ± s.e.m. (*n* = 4 (control) or 5 (compound) tumors for each arm of the U-CH1/RMC-4550 study; *n* = 6 (compound) or 7 (control) tumors for each arm of the U-CH1/TNO155 study; *n* = 6 (control) or 7 (compound) tumors for each arm of the CF539 study; *n* = 7 tumors for each arm of the CF466 study). n.s., not significant, *P < 0.05, ****P < 0.0001, derived from a two-way analysis of variance (ANOVA) with repeated measures. *P* values for the time × treatment interaction (relative to the control condition) are indicated. Additional details of *P* values and effect sizes are reported in Supplementary Data 7. Source data are provided as a Source Data file. See also related Supplementary Data 6.

Six chordoma cell lines each were treated with allosteric compounds targeting SHP2 (RMC-4550 or SHP099) and assayed for cell viability (Fig. 5a, b). For comparison, we also tested the *BRAF*^V600E^-mutant A2058 melanoma cell line and the MDA-MB-468 breast adenocarcinoma cell line, which are reported to be insensitive and sensitive to SHP2 inhibition, respectively^47^, and which accordingly exhibit differential dependence on *PTPN11* gene suppression (Supplementary Fig. 10). All cell lines were subjected to both 14-d colony formation and 6-day multipoint concentration-response assays, the former of which better distinguished the sensitivity of control cell lines (Fig. 5a, b). We observed that five of the six chordoma cell lines tested were sensitive to SHP2 inhibition, to a degree exceeding that observed for the MDA-MB-468 reference cell line (Fig. 5a, b; area-under-curve (AUC) and half-maximal effective concentration (EC_50_) values reported in Supplementary Data 6). We confirmed on-target activity for SHP2 inhibitors by detecting reduced phosphorylation of ERK 1/2 in sensitive cell lines, with RMC-4550 exhibiting more potent on-target activity than SHP099 (Fig. 5c).

To test the efficacy of SHP2 inhibitors in vivo, a U-CH1-derived xenograft mouse model of chordoma was treated with 10 or 30 mg/kg RMC-4550 or vehicle. The selected doses of RMC-4550 were determined to be capable of reducing phosphorylation of ERK 1/2 in the tumors of treated animals (Supplementary Fig. 11). Consistent with ex vivo findings, when dosed for 21 days, RMC-4550 inhibited tumor growth compared to vehicle treatment (Fig. 5d and Supplementary Fig. 12), with the 30 mg/kg dose meeting a threshold of significance (*P* = 0.044 for 30 mg/kg; *P* = 0.060 for 10 mg/kg). Alongside experiments with the tool compound RMC-4550, we tested a clinical-stage SHP2 inhibitor in this model to further evaluate the potential for clinical translation of these findings. Consistent with the RMC-4550 study, statistically significant antitumor efficacy was achieved in the U-CH1 mouse model treated with 20 mg/kg of TNO155 (*P* = 1.27 × 10^−6^), a clinical-stage SHP2 inhibitor with ex vivo anti-chordoma activity comparable to that of RMC-4550 and SHP099 (Fig. 5d and Supplementary Figs. 12, 13).

TNO155 was also tested in two patient-derived xenografts (PDX) models, representing distinct patient populations of chordoma: CF539 was derived from a pediatric metastatic clival chordoma, and CF466 was derived from an adult metastatic lumbar chordoma. TNO155 treatment led to significant suppression or regression of chordoma tumor proliferation in these models, with a good tolerability profile (Fig. 5d and Supplementary Fig. 12, 14). Tumor growth suppression induced by TNO155 treatment showed features of growth arrest, but not apoptotic cell death (Supplementary Fig. 14). Together, these findings using small-molecule perturbation of SHP2 further corroborate the essentiality of *PTPN11* in various models of chordoma, and thus nominate SHP2 inhibitors as a therapeutic approach for the treatment of chordoma.

## Discussion

Here we describe the emerging landscape of selective dependencies in chordoma. This study recovered the most significant known dependency gene in chordoma, *TBXT*, and further revealed a spectrum of additional selectively essential genes in this cancer type. These genes included several targetable or potentially targetable vulnerabilities that would not otherwise be detected by tumor sequencing approaches.

We note that while we focused our study on selectively essential genes, hypothesizing that these dependencies could be exploited to target chordoma while minimizing toxicity to normal tissue, other essential genes may warrant further exploration. For example, some genes that are highly essential in but have lower selectivity scores for chordoma, such as those encoding CDK7, CDK9, and GPX4—proteins against which small-molecule inhibition was previously shown to have antiproliferative effects in chordoma models^17^ – may represent cancer targets with potential relevance to diverse cancer types including chordoma (Supplementary Fig. 15)^49,50^.

**Fig. 6:**
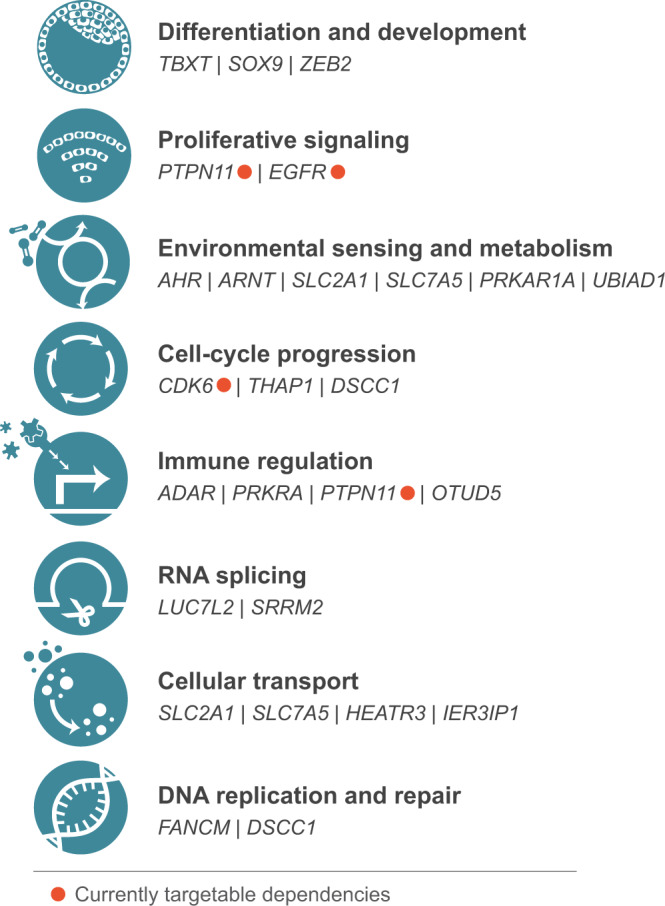
Functional classification of chordoma dependency genes. Canonical biological functions associated with chordoma dependency genes. Genes encoding proteins that are currently targetable are indicated.

Collectively, the dependency genes identified herein can be classified into a broad range of biological functions. Canonical roles for these genes include: differentiation and development (the developmental transcription factors *TBXT*, *SOX9*, and *ZEB2*); proliferative signaling (*PTPN11* and *EGFR*); environmental sensing and metabolism (the aryl hydrocarbon receptor *AHR* and its transcription partner *ARNT*, the glucose transporter *SLC2A1*, the amino-acid transporter *SLC7A5*, the cAMP signaling regulatory component *PRKAR1A*, and the cholesterol metabolism regulator *UBIAD1*); cell-cycle progression (the cell cycle cyclin-dependent kinase *CDK6*, a cell-cycle gene regulator *THAP1*, and regulator of cell-cycle checkpoint activation and DNA replication *DSCC1*)^51,52^; immune regulation (*ADAR*, *PRKRA*, *PTPN11*, and a suppressor of the innate immune system *OTUD5*); RNA splicing (the U1 snRNP subunit gene *LUC7L2*, and a pre-mRNA splicing co-activator *SRRM2*)^53^; cellular transport (*SLC2A1*, *SLC7A5*, the ribosomal protein transport-related gene *HEATR3*, and a regulator of endoplasmic reticulum and secretory function *IER3IP1*)^54,55^; and DNA replication and repair (the Fanconi anemia core complex component *FANCM*, and *DSCC1*) (Fig. 6).

We observed several instances of non-oncogene dependencies, whereby the unique cellular features of the cancer cell confer a heightened dependence on the normal function of specific genes^12^. For example, chordoma cells’ high intrinsic expression of ISGs appears to underlie their dependence on the double-strand RNA (dsRNA)-editing enzyme *ADAR*. It has been proposed that a chronic ISG state confers dependence on *ADAR* because it poises nucleic acid sensors like PKR to respond to the accumulation of endogenous immunogenic dsRNAs that *ADAR* normally edits^36,37,40^. Further investigation using additional models is needed to understand what triggers chronic ISG expression in chordoma and whether this is a frequently occurring feature of cancer. In other cancer types, the STING cytosolic DNA sensing pathway has been implicated in IFN-induced ISG expression, suggesting a link between chronic ISG expression and genomic instability^37,56^. Notably, recent evidence suggests that chordoma tumors frequently exhibit characteristics of defective homologous recombination DNA repair and increased genomic instability^57^; and some chordoma tumors harbor large numbers of clustered genomic rearrangements consistent with the phenomenon of chromothripsis^16,58^, features each of which is associated with the accumulation of immunostimulatory DNA^56,59^. It remains to be determined whether these elements contribute to chordoma cells’ high expression of ISGs and consequent dependence on *ADAR*.

Another dependency gene whose essentiality correlates with a specific cellular feature is *LUC7L2*. Here, dependency is correlated with a lower copy number of a region containing the *LUC7L2* paralog *LUC7L*. This interaction is suggestive of a paralog lethality, whereby the loss of one member of a paralog pair is associated with increased reliance on the other member^7,8^. These findings are consistent with that of a recent report demonstrating cross-regulation and partial redundancy between *LUC7L2* and *LUC7L*^60^.

Additionally, two genes scoring in our screens (*TBXT*, *SOX9*) can be classified as lineage-survival dependency genes: these transcription factors mediate normal development of the embryonic notochord, the cell type from which chordoma is hypothesized to originate^61–63^. We also noted that three of the genes identified as chordoma dependency genes (*THAP1*, *SLC2A1*, and *PRKRA*) are causative for dystonia, a neurological condition characterized by involuntary muscle contractions^64^. Further investigation is required to determine whether these results indicate a related etiology and/or common cell lineage from which chordoma and dystonia arise.

Lastly, our findings nominate an immediately actionable therapeutic target, SHP2, for the treatment of chordoma. Previous work has shown that cell lines dependent on *PTPN11* are most dependent on *EGFR*^47^. Our screens in chordoma similarly demonstrate dependency on both genes, but we noted that *PTPN11* dependency scores are of a greater magnitude than those of *EGFR* (Fig. 1b, c and Supplementary Fig. 2). Further study is required to understand the mechanism underlying *PTPN11* dependency in chordoma; for example, it is possible that SHP2 inhibition attenuates signaling mediated by different growth factor receptors, several of which can be activated concurrently in chordoma^65^. First-generation SHP2 inhibitors like TNO155 are currently under clinical evaluation (e.g., ClinicalTrials.gov identifier: NCT03114319); chordoma cells’ differential sensitivity to SHP2 suppression relative to other cancer types supports testing these inhibitors in chordoma patients.

## Methods

This study complies with all relevant ethical regulations.

### Cell lines and reagents

UM-Chor1, JHC7, MUG-Chor1, U-CH1, and U-CH2 chordoma cell lines were obtained from the Chordoma Foundation. CH22 cells have been described previously in ref. ^66^ and were obtained from the Chordoma Foundation and Massachusetts General Hospital. MDA-MB-468 breast adenocarcinoma and A2058 melanoma cell lines were obtained from ATCC. UM-Chor1 cells were maintained in IMDM/RPMI (4:1) media + 10% fetal bovine serum (FBS) and 1X non-essential amino acids. JHC7 cells were maintained in DMEM/F12 (1:1) + 10% FBS. MUG-Chor1, U-CH1, and U-CH2 cell lines were maintained in IMDM/RPMI (4:1) media + 10% FBS. CH22 and MDA-MB-468 cells were maintained in RPMI media + 10% FBS. A2058 cells were maintained in DMEM media + 10% FBS. All chordoma cell lines were maintained on collagen I-coated plates.

RMC-4550 and SHP099 were purchased from MedChemExpress. TNO155 used for ex vivo studies was purchased from Selleck Chemicals, and that used for in vivo studies was purchased from MedChemExpress.

### Genome-scale CRISPR-Cas9 screening

Genome-scale CRISPR-Cas9 screens using UM-Chor1 and MUG-Chor1 cell lines were performed previously^17^.

For U-CH2 and JHC7 cell lines, Cas9-expressing cells were generated as follows: each parental cell line was incubated with lentivirus corresponding to the pLX_311-Cas9 plasmid (Addgene plasmid #96924), encoding the Cas9 protein, in the presence of 4 μg/mL polybrene, dispensed in 12-well plates (1.5 × 10^6^ cells/well), and spin-infected at 2000 rpm for 2 h at 30 °C. After spin-infection, 2 mL of standard growth media was added to each well, and cells were incubated at 37 °C overnight. The following day, for each cell line, cells were trypsinized and expanded in selective media containing 2–3 µg/mL blasticidin. Following selection for infected cells, Cas9 activity was confirmed in each transduced cell line using a Cas9-activity assay that has been described previously in ref. ^67^.

Genome-scale screens were performed using a library of 74,687 unique sgRNAs targeting ~18,560 genes (typically four sgRNAs per gene) and 1000 non-targeting control sgRNAs (Broad Institute Avana sgRNA library)^18^. U-CH2-Cas9 and JHC7-Cas9 cells were each incubated with lentivirus corresponding to the pooled CRISPR library in the presence of 4 μg/mL polybrene, dispensed in 12-well plates (1.5 × 10^6^ cells/well across 21 plates for U-CH2 and 24 plates for JHC7), and spin-infected at 2000 rpm for 2 h at 30 °C. Lentivirus was titered in each cell line to achieve a low MOI (<1), and infections were performed with a sufficient number of cells to achieve a representation of >700 cells per sgRNA per replicate after selection for infected cells. Following spin-infection, 2 mL of standard growth media was added to each well, and cells were incubated at 37 °C overnight. The next day, for each cell line, cells were trypsinized, divided into three replicates, and expanded in selective media containing puromycin (14 µg/mL for U-CH2 and 5 µg/mL for JHC7) and blasticidin (3 µg/mL for U-CH2 and 1 µg/mL for JHC7). Cells were grown in culture for 20 days (U-CH2) or 22 days (JHC7) post-infection, with carryover of 5 × 10^7^ (U-CH2) or 4 × 10^7^ (JHC7) cells at each passage. Cells were grown in selective media until 8 days (JHC7) or 13 days (U-CH2) post-infection, after which they were grown in standard growth media. At 20 days (U-CH2) or 22 days (JHC7) post-infection, cells were collected and stored at −20 °C in PBS until genomic DNA isolation steps.

Genomic DNA was isolated from cell pellets using the NucleoSpin Blood XL Kit (Macherey-Nagel). The sgRNA sequence was amplified by PCR with sufficient gDNA to maintain representation, and then quantified using massively parallel sequencing^68,69^. For each cell line, primary screening was performed once with three replicates.

### Computational analysis software for genetic studies

Unless otherwise stated, all genetic-perturbation analyses were performed in R (version 4.0.2) using the tidyverse package (version 1.3.0).

### Analysis of genome-scale CRISPR-Cas9 screening data

The sgRNA library we used to screen the four chordoma cell lines described in this study (Broad Institute Avana sgRNA library)^18^ has also been used to screen hundreds of non-chordoma cancer cell lines by the Broad Institute Cancer Dependency Map (DepMap) project (https://depmap.org/portal/). Thus, we were able to combine our data with the DepMap data. Specifically, we combined read count data from four chordoma cell lines (JHC7, U-CH2, UM-Chor1, and MUG-Chor1) with those from 765 non-chordoma cancer cell lines. Readcount data from all cell lines were normalized and subjected to quality control checks, processed by CERES, and post-processed in the standard pipeline described previously in ref. ^19^, resulting in the CERES gene effect and dependency probability scores used in our analysis. The combined data for all 769 chordoma and non-chordoma cell lines used in this study are available through the 20Q2 DepMap release (https://depmap.org/portal/download/; 10.6084/m9.figshare.12280541.v4).

We favored CERES (to generate gene effect scores, from which dependency probability scores were also derived) because it takes advantage of screening data generated across a large collection of cell lines to infer sgRNA activity^18,19^. Moreover, CERES corrects for gene-independent lethality associated with CRISPR-Cas9 targeting of copy number-amplified regions, a known confounder of CRISPR-Cas9 essentiality screens^18^.

We obtained genomic and transcriptomic data (gene-expression values, copy-number variations, and mutations) for all non-chordoma cell lines from the 20Q2 DepMap release. Genome and transcriptome sequencing data for chordoma cell lines were provided by the Chordoma Foundation (available at http://www.cavatica.org/). Transcriptomic data for chordoma cell lines were processed as described below. Genomic data for chordoma cell lines were processed by and shared through the DepMap portal (see DepMap release notes for details; https://depmap.org), and are available from the 20Q2 DepMap release, except mutation calls for U-CH2, which are available from the 22Q1 DepMap release (10.6084/m9.figshare.19139906.v1).

To identify selective dependencies in chordoma, we compared gene dependency scores between the four chordoma lines and 765 non-chordoma cell lines using a linear model implemented in the R package limma (version 3.44.3)^70^. We performed this differential essentiality analysis for both CERES and dependency probability scores. The difference in mean dependency between the chordoma and non-chordoma lines was evaluated per gene as a log_2_ fold-change, and associated *P* values were derived from empirical-Bayes-moderated t-statistics. *Q* values were computed using the Benjamini–Hochberg method^71^.

Based on this analysis, selective dependencies were nominated using both CERES and dependency probability score statistics. We aimed to be inclusive in selecting dependency genes for validation. We, therefore, started with stringent thresholds (*P* < 0.01, absolute log_2_ fold-change >0.5 for both CERES gene effect and dependency probability scores), yielding three selective dependencies. We then relaxed these thresholds to select the final set of 21 chordoma selective dependencies: genes were considered selective if (1) their CERES differential *P* value was lower than 0.02, (2) their absolute log_2_ fold-change in CERES scores exceeded 0.4, and (3) their absolute log_2_ fold-change in dependency probability exceeded 0.3. We note that the *P* value threshold is relatively robust to small changes. A threshold of *P* < 0.05 would have nominated the same set of chordoma-selective dependencies. In addition, genes selective for chordoma needed to exceed an average dependency probability for chordoma cell lines of 0.5 and have a positive log_2_ fold-change (chordoma vs. non-chordoma). By contrast, genes selective for other cancer types needed to have an average dependency probability for chordoma cell lines equal to or lower than 0.5, with a negative log_2_ fold-change (chordoma vs. non-chordoma).

### Lentiviral vectors used for screening validation and functional characterization

To validate primary screening results, for each gene of interest, two sgRNA sequences represented in the Broad Institute Avana sgRNA library were selected and cloned into the lentiGuide-Puro plasmid (Addgene #52963). The spacer sequence for sg-*EGFP* has been described previously (“EGFP sgRNA 6”)^72^.

Spacer sequences for sgRNAs were as follows:

**Table:** 

Target gene guide	Spacer sequence
sg-*ADAR*−1	ACAATGGCCCCTCAAAAGCA
sg-*ADAR*−2	ACTCCAAAAGGCCACCCACA
sg-*CDK6*−1	AAGGCCCGCGACTTGAAGAA
sg-*CDK6*−2	CCAGCAGTACGAATGCGTGG
sg-*EGFP*	GGTGAACCGCATCGAGCTGA
sg-*FANCM*−1	CCTTTCCTGAAGGGAACCAG
sg-*FANCM*−2	GCATAAGGCCTATAAAATGG
sg-*LUC7L2*−1	CCATGACCTGGCTTTAAGAG
sg-*LUC7L2*−2	GGATGAAGTAGAGAAAGCAC
sg-*PRKRA*−1	AGATGATAACAGCTAAGCCA
sg-*PRKRA*−2	TTCACCTTCAGAGTAACCGT
sg-*PTPN11*−1	GGAGGAACATGACATCGCGG
sg-*PTPN11*−2	GTGCAGATCCTACCTCTGAA
sg-*SLC2A1*−1	AGTGTTGTAGCCAAACTGCA
sg-*SLC2A1*−2	GGAGTTCTACAACCAGACAT
sg-*SLC7A5*−1	CGGAACATCACGCTGCTCAA
sg-*SLC7A5*−2	GATGCTGGCCGCCAAGAGCG
sg-*SOX9*−1	GCACCTGGCTGACCGCCTCG
sg-*SOX9*−2	GCTGGTACTTGTAATCCGGG
sg-*SRRM2*−1	GCAGGTCTCTCTCTTCACCA
sg-*SRRM2*−2	GCATGCCGAGAAACTTTGGT
sg-*THAP1*−1	GTGCAGTCCTGCTCCGCCTA
sg-*THAP1*−2	CCTCACTTGTGGAAAGAAAC

Lentivirus was produced by transfection of 293T packaging cells with three plasmids (lentiGuide-Puro-sgRNA, psPAX2, and pMD2.G); and the Lipofectamine 2000 transfection reagent (Invitrogen). Media was changed to standard growth media the following day, and virus-containing supernatant was collected 3 days post-transfection.

### Validation of genome-scale CRISPR-Cas9 screens

UM-Chor1-Cas9 cells were generated as described previously in ref. ^17^. UM-Chor1-Cas9 cells were seeded at a density of 300,000 cells/well in six-well collagen I-coated plates. The next day, media was replaced with media containing lentivirus corresponding to the lentiGuide-Puro plasmid (Addgene #52963), encoding the sgRNA of interest (sequences below), in the presence of 8 µg/mL polybrene. Cells were spin-infected at 2000 rpm for 30 min at 30 °C. Following spin-infection, media was replaced with standard growth media and cells were incubated at 37 °C overnight. The next day (at least 24 h post-infection), the media was replaced with selective media containing 1 µg/mL puromycin. Following selection for infected cells, cells were seeded in 24-well plates in media containing 1 µg/mL puromycin at a density of 30,000 cells/well (three replicates per timepoint) and counted at indicated intervals over a 10-day period. Cell counts were obtained with a Vi-CELL XR Cell Viability Analyzer (Beckman Coulter), using Vi-CELL XR software (version 2.04; Beckman Coulter). Proliferation experiments were done four times for guides targeting *PTPN11*, *CDK6*, and *SLC7A5*; and twice for guides targeting all other genes, with minor variations in time intervals used for counting. Statistical analyses were performed with GraphPad Prism 9.

For immunoblotting to confirm sgRNA-mediated protein reduction, cells were transduced as above and selected cells were harvested 7 days post-transduction. Cell pellets were subjected to immunoblotting as described below. The experiment was performed three times for *PTPN11*; twice for *SLC7A5*, *PRKRA*, and *LUC7L2*; and once for other genes.

For amplicon sequencing to confirm sgRNA-mediated genomic editing, cells were transduced as above and selected cells were harvested 6 days post-transduction. Cell pellets were processed as described below. The experiment was performed once.

### Immunoblotting

Cell pellets were resuspended in lysis buffer (50 mM Tris pH 7.4, 2.5 mM EDTA pH 8, 150 mM NaCl, 1% Triton X-100, and 0.25% IGEPAL CA-630) supplemented with protease inhibitors (Roche) and Phosphatase Inhibitor Mixtures I and II (Calbiochem). Lysates were incubated on ice for >2 min, then centrifuged for 2 min at 15,700 × *g*. Protein in the supernatants was quantified using a BCA Protein Assay Kit (Pierce), normalized, reduced, and denatured. Protein samples were resolved using Tris-Glycine gels (Novex), then resolved protein was transferred to iBlot Transfer Stack nitrocellulose membranes (Novex). Membranes were probed with primary antibodies at 4 °C overnight. The antibodies against SHP2 (clone D50F2, no. 3397, 1:1000), total Erk1/2 (clone 3A7; no. 9107, 1:500), phospho-Erk1/2 (Thr202/Tyr204) (no. 9101, 1:500), CDK6 (clone D4S8S, no. 13331, 1:500), PRKRA/PACT (clone D9N6J; no. 13490; 1:1,000), SLC7A5/LAT1 (no. 5347, 1:1,000), cofilin (clone D3F9, no. 5175; 1:10,000), and SOX9 (clone D8G8H, no. 82630, 1:1,000) were purchased from Cell Signaling Technology. The antibody against LUC7L2 (no. PA5-62446, 1:1,000) was purchased from Invitrogen. Membranes were incubated with IRDye secondary antibodies (1:10,000; LI-COR Biosciences), and immunoblot images were acquired with the Odyssey Imaging System (LI-COR Biosciences), using Image Studio software (version 2.0.38; LI-COR Biosciences).

### Amplicon sequencing of sgRNA-mediated genomic edit sites

Genomic DNA was purified from cell pellets using the QIAamp DNA Mini Kit (Qiagen). A 200–275 base pair region containing the relevant sgRNA target sequence was PCR-amplified from genomic DNA using the NEBNext High-Fidelity 2X PCR Master Mix (New England BioLabs) in a 25 µL PCR reaction volume (primer sequences are provided in Supplementary Table 2). PCR products were purified using the QIAquick PCR purification Kit (Qiagen).

Library preparation and sequencing were performed at the Dana-Farber Cancer Institute Molecular Biology Core Facilities. cDNA amplicons were fragmented to ~250 base pairs using Covaris adaptive focused acoustics on the M220 platform. Illumina sequencing libraries were prepared using Swift S2 Acel reagents on a Biomek i7 liquid handling platform. Finished libraries were quantified by Qubit fluorometer, Agilent TapeStation 2200, and RT-qPCR using the Kapa Biosystems library quantification kit according to the manufacturer’s protocols. Uniquely indexed libraries were pooled in equimolar ratios and sequenced on an Illumina MiSeq with paired-end 150-base pair reads.

Amplicon sequencing was performed once.

### Amplicon sequencing analysis

We evaluated the editing performance of sgRNAs from amplicon sequencing data using CRISPResso2 (version 2.1.3)^73^ with default parameters. Gene reference sequences were obtained from the NCBI Nucleotide database using the R package rentrez (version 1.2.3) and expected amplicon sequences were extracted with the matchProbePair function from the R package Biostrings (version 2.56.0). Potential off-target genomic sequences were identified with NCBI BLAST (https://blast.ncbi.nlm.nih.gov/Blast.cgi) and provided to CRISPResso2 as alternate sequences to reduce artifactual reporting of editing.

### Pathway analysis of chordoma dependency genes

#### Co-essentiality network

The network was generated using the R package igraph (version 1.2.6). First, all selective chordoma dependency genes were added as nodes. To draw edges between gene pairs, we calculated the Pearson correlation coefficient between their dependency probability score profiles (dependency probability scores for all 769 cell lines). Gene pairs with a correlation of least 0.18 were connected by an edge. We visualized the resulting network in Cytoscape (version 3.8.2), coloring nodes by dependency probability scores and scaling edge width by Pearson correlation coefficients.

#### Protein–protein interaction network

We performed a multi-protein search in STRING (version 11.5) (https://string-db.org/)^74^ with default parameters, using HGNC symbols for all selective chordoma dependency genes as input. The resulting network was exported and visualized in Cytoscape (version 3.8.2), coloring nodes by dependency probability scores and scaling edge width by STRING confidence scores. Singletons, removed automatically during export from STRING, were manually re-added in Cytoscape.

### RNA-sequencing analysis of parental chordoma cells

Gene-expression levels of chordoma cell lines were quantified from RNA-seq data using the DepMap RNA-seq pipeline (https://github.com/broadinstitute/depmap_omics) on the Terra computing platform (https://terra.bio/). Briefly, RNA-seq FASTQ files were aligned to the hg38 reference genome (Ensembl; https://useast.ensembl.org/Homo_sapiens/Info/Index) with STAR (version 2.5.3a)^75^. Gene- and transcript-level expression was then quantified with RSEM (version 1.3.0)^76^ to obtain transcript per million (TPM) values.

### Uniform manifold approximation and projection (UMAP) analysis

To generate two-dimensional UMAP embeddings, we used log_2_(TPM + 1) expression values for all genes whose coefficient of variation exceeded 0.1 across all 1299 cell lines analyzed. The top 100 principal components of the resulting data set were then used as input to the umap function of the umap R package (version 0.2.8.0) with the following parameter settings: n_neighbors = 5, min_dist = 0.5, n_epochs = 500. Default settings were used for all other parameters. Cell lines without disease annotation, disease annotations with only one cell line (teratoma and adrenal cancer), and the disease annotation “Engineered” were omitted from the analysis.

### Genomic and transcriptomic correlates of chordoma dependency genes

For all chordoma dependency genes, we calculated Pearson correlation coefficients between their CERES dependency profiles (CERES scores for a given gene across all cell lines) and genome-wide (1) gene-expression profiles (gene-level log_2_(TPM + 1) values across all cell lines), (2) copy-number profiles (gene copy number across all cell lines), and (3) mutation profiles (gene mutations across all cell lines, indicating the presence or absence of mutations as 1 and 0, respectively). Furthermore, we performed univariate linear regressions, predicting CERES gene effect scores for each chordoma dependency while using each individual genomic/transcriptomic feature as a predictor.

For dependencies correlated with gene-copy-number changes, we nominated potentially causal genes based on known biological connections to the dependency gene. We further tested whether the dependency also correlated with the copy number of genes surrounding the nominated gene on the same chromosome. To do so, we first selected all genes within windows ranging from 5 × 10^5^ bp to 5 × 10^6^ bp (step size: 5 × 10^5^) around the nominated gene. The optimal window was then determined using gene-set enrichment analysis^41^ via the fgsea function from the synonymous R package^77^ (settings: eps = 0, the default for all others). As input, we used (1) the gene sets obtained from each window size and (2) the list of all gene-copy-number correlates, ranked by their correlation with the dependency gene.

### ISG expression in chordoma and non-chordoma cancer cell lines

A previously described procedure^37^ was followed using the ISG core signature from the same study (*ADAR*, *BST2*, *CASP1*, *CMPK2*, *CXCL10*, *DDX60*, *DHX58*, *EIF2AK2*, *EPSTI1*, *GBP4*, *HERC6*, *IFI35*, *IFIH1*, *IFIT2*, *IFIT3*, *IRF7*, *ISG15*, *ISG20*, *MX1*, *NMI*, *OASL*, *OGFR*, *PARP12*, *PARP14*, *PNPT1*, *PSME2*, *RSAD2*, *RTP4*, *SAMD9L*, SP1*10*, *STAT2*, *TDRD7*, *TRAFD1*, *TRIM14*, *TRIM21*, *TRIM25*, *UBE2L6*, and *USP18*). Briefly, mean absolute deviation modified *z*-scores (ZMAD) were calculated for each gene, using median and mean absolute deviation for TPM values across all cell lines. The ISG core score was then calculated as the mean ZMAD across all ISG signature genes. Cell lines without disease annotation, disease annotations with only one cell line (teratoma and adrenal cancer), and the disease annotation “Engineered” were omitted from the analysis. We repeated this procedure with two additional interferon signatures from the MSigDB hallmark collection^39^ (version 7.4) (http://www.gsea-msigdb.org/gsea/msigdb/human/genesets.jsp?collection=H): the gene sets HALLMARK_INTERFERON_ALPHA_RESPONSE (http://www.gsea-msigdb.org/gsea/msigdb/human/geneset/HALLMARK_INTERFERON_ALPHA_RESPONSE.html) and HALLMARK_INTERFERON_GAMMA_RESPONSE (http://www.gsea-msigdb.org/gsea/msigdb/human/geneset/HALLMARK_INTERFERON_GAMMA_RESPONSE.html).

### RNA sequencing and analysis of sgRNA-treated cells

UM-Chor1-Cas9 cells were seeded at a density of 300,000 cells/well in six-well collagen I-coated plates. Cells were transduced with lentivirus and selected for infected cells as described in the Methods section corresponding to CRISPR-Cas9 screening validation. Following selection for infected cells, selective growth media was replaced with standard growth media. One day post-media-change (6 days post-transduction), cells were harvested. Total RNA was extracted from cells using an RNeasy kit (Qiagen).

Library preparation, sequencing, and sequencing analysis were performed at the Dana-Farber Cancer Institute Molecular Biology Core Facilities. Libraries were prepared using Roche Kapa mRNA HyperPrep strand-specific sample preparation kits from 200 ng of purified total RNA according to the manufacturer’s protocol on a Beckman Coulter Biomek i7. The finished dsDNA libraries were quantified by Qubit fluorometer and Agilent TapeStation 4200. Uniquely dual indexed libraries were pooled in an equimolar ratio and shallowly sequenced on an Illumina MiSeq to further evaluate library quality and pool balance. The final pool was sequenced on an Illumina NovaSeq 6000, targeting 40 million 100-base pair read pairs per library.

Sequenced reads were aligned to the UCSC hg38 reference genome assembly (http://genome.ucsc.edu/cgi-bin/hgGateway?db=hg38), and gene counts were quantified using STAR (version 2.7.3a)^75^. RNA-seq analysis was performed using the VIPER Snakemake pipeline^78^. Differential gene-expression testing was performed by DESeq2 (version 1.22.1)^79^.

We performed gene-set enrichment analysis^41^ using the fgsea function from the synonymous R package^77^ (settings: eps = 0, the default for all others) on the MSigDB hallmark collection of gene sets (version 7.4)^39^, with log_2_ fold-changes from DESeq2 as input.

We used GeLiNEA to quantify enrichment while taking biological network information into account^42^. We ran GeLiNEA via the Molecular Data Provider (MolePro) API (https://translator.broadinstitute.org/molecular_data_provider/assets/lib/swagger-ui/index.html?url=/molecular_data_provider/assets/openapi.json#/), using the MSigDB hallmark collection of gene sets and the STRING interaction network (version 10.5, *Homo sapiens*, including all available evidence types)^74^. The source code for GeLiNEA can be obtained at https://github.com/broadinstitute/GeLiNEA. The API calls were made using a MATLAB script (2018b version), with the parallelization toolbox for parallelizing the calls.

Rather than a ranked list, GeLiNEA requires a list of top-scoring genes as input (in addition to a list of known gene sets). To account for differences in list size, we considered all lists from 1 to 200 top-scoring genes, with gene ranks determined by their adjusted *P* value from the previously described DESeq2 differential-expression analysis. Results were robust against list-size variation, indicating strong enrichment of interferon gene sets among the most upregulated genes. Using a MATLAB 2018b implementation, we then constructed curves charting gene list size against the negative log_10_ of adjusted enrichment *P* values, and ranked hallmark gene sets based on their normalized AUC, calculated as follows:

1 $${{{{{\rm{AUC}}}}}}=\frac{1}{200*{p}_{\min }}.\,{\int }_{g=1}^{200}-{{{{{{\rm{log }}}}}}}_{10}{p}_{{{{{{{\mathrm{adj}}}}}}}}\left(g\right).{dg}$$

where ${p}_{\min }=\mathop{{{{{{\rm{lim}}}}}}}\nolimits_{n\to+\infty }-{{{{{{\rm{log }}}}}}}_{10}({p}_{{{{{{{\mathrm{adj}}}}}}}})=-{{{{{{\rm{log }}}}}}}_{10}0+\varepsilon=52$, and ε equals the inverse logarithm base 10 of the floating-point relative accuracy.

RNA sequencing was performed once.

### IFN-β ELISA

UM-Chor1-Cas9 cells were seeded at a density of 300,000 cells/well in six-well collagen I-coated plates. Cells were transduced with lentivirus as described in the Methods section corresponding to CRISPR-Cas9 screening validation. Following selection for infected cells with media containing 2 µg/mL puromycin, selective growth media was replaced with standard growth media. At 1, 2, and 3 days post-media-change, an aliquot of conditioned media was harvested from cells and centrifuged to remove any residual cells. The supernatant was assayed for IFN-β levels using the VeriKine-HS™ Human IFN Beta Serum ELISA Kit (PBL Assay Science), following “Protocol A” provided by the manufacturer.

Absorbance values of the ELISA were measured at 450 nm with a SpectraMax M5 microplate reader (Molecular Devices), using SoftMax Pro software (version 5.4; Molecular Devices). To calculate IFN-β titers, optical densities (ODs) for media alone samples were first subtracted from the standard and sample ODs to eliminate background, and the IFN-β concentration in the samples was determined from a standard curve, fit with a sigmoidal, four-parameter logistic equation (GraphPad Prism 9). Concentrations too low to be determined by the standard curve were set to zero. Statistical analyses were performed with GraphPad Prism 9. The experiment was performed three times.

### Computational analysis software for small-molecule sensitivity studies

Computational analyses and visualizations for small-molecule studies were performed in Excel from Microsoft 365 (version 2301 and predecessors; Microsoft Corp.), Pipeline Pilot (version 18.1.0.1604; Biovia, Corp.), or MATLAB 2018b (MathWorks, Inc.).

### High-throughput small-molecule and IFN-β sensitivity studies

For small-molecule sensitivity studies, U-CH1, U-CH2, JHC7, MUG-Chor1, UM-Chor1, CH22, MDA-MB-468, and A2058 cells were each seeded overnight in 384-well BioCoat Collagen I (Corning) microtiter plates at a density of 2000, 1600, 1600, 1800, 1200, 1200, 1200, and 1000 cells per well, respectively. The following day, compound or DMSO was added to wells with a Tecan D300e digital dispenser instrument (HP) using Tecan D300eCONTROL software (version 3.2.5; HP). Each compound was tested using nine concentrations in quadruplicate (four wells treated in parallel). Cell viability was assayed 6 days after compound addition with the CellTiter-Glo reagent (Promega). Luminescence values were acquired with an EnVision microplate reader (PerkinElmer), using EnVision Manager software (versions 1.13.3009.1401 and 1.14.3049.528; PerkinElmer). Luminescence well values were normalized to DMSO-treated wells by subtracting per-plate average DMSO log_2_-luminescence values from the log_2_-luminescence values of each treatment well.

For IFN-β sensitivity studies, UM-Chor1 cells were seeded overnight in 384-well BioCoat Collagen I (Corning) microtiter plates at a density of 1200 cells per well. The following day, recombinant IFN-β (PBL Assay Science human IFN-beta 1a, #11415) or vehicle was added to wells in quintuplicate (five wells treated in parallel). Cell viability was assayed 6 days after IFN-β addition with the CellTiter-Glo reagent (Promega). Luminescence values were acquired with an EnVision microplate reader (PerkinElmer), using EnVision Manager software (version 1.14.3049.528; PerkinElmer). Luminescence well values were normalized to vehicle-treated wells by subtracting per-plate average vehicle log_2_-luminescence values from the log_2_-luminescence values of each treatment well.

Data pre-processing from instrument files through DMSO/vehicle normalization was performed in Pipeline Pilot except for the experiments depicted in Supplementary Figs. 9, 13, which were normalized in Microsoft Excel and MATLAB; curve-fitting, numerical integration, and subsequent analysis steps were performed in MATLAB. For all drug-printer experiments, curves were fit using all data points as inputs to curve-fitting and numerical integration. Curve fitting (to derive EC_50_ and other curve parameters) and numerical integration (to derive AUC) were otherwise performed as described previously^17^.

SHP099 was tested five times in UM-Chor1 cells (including in the experiment depicted in Supplementary Fig. 13), twice in JHC7, and once in all other cell lines. RMC-4550 was tested four times in UM-Chor1 (including in the experiment depicted in Supplementary Fig. 13), twice in JHC7, and once in all other cell lines. TNO155 was tested twice in UM-Chor1 cells. IFN-β was tested three times in UM-Chor1 cells.

### Colony formation assays

U-CH1, U-CH2, JHC7, MUG-Chor1, UM-Chor1, CH22, MDA-MB-468, and A2058 cells were seeded in six-well plates at a density of 70,000, 50,000, 80,000, 90,000, 18,000, 6,000, 15,000, and 2000 cells/well, respectively. The following day, RMC-4550 or DMSO was added to wells at a 1:1000 dilution. Cells were cultured in a compound- or DMSO-containing media for a total of 14 days, with the compound- or DMSO-containing media replenished at 7 days post-treatment. At the experimental endpoint, compound- or DMSO-containing media was aspirated, and cells were first washed with PBS, then fixed with 100% methanol for 10 min. Methanol was aspirated and cells were stained with 0.5% crystal violet (Alfa Aesar) staining solution in 25% methanol for 10 min. The staining solution was aspirated and plates were washed with water and subsequently air dried. Each cell line was tested at least four times, with minor variations in cell seeding densities.

Plates were imaged with an Epson Perfection V550 Photo scanner.

### Immunoblots of compound-treated cell lines

For immunoblots of SHP2-inhibitor-treated cell lines: for each cell line, cells were seeded in a six-well plate at a density of 400,000 cells/well. The following day, cells were treated with RMC-4550, SHP099, or DMSO (1:1000 dilution in media) for 2 h before being harvested. Cell pellets were lysed and processed for immunoblotting as described above. The experiment was performed at least twice for UM-Chor1 and JHC7, and once for all other cell lines.

### *PTPN11* dependency in MDA-MB-468 and A2058 cell lines

Data corresponding to *PTPN11* log_2_(TPM + 1) expression (21Q3 Public) and *PTPN11* gene effect by CRISPR (DepMap 21Q3 Public+Score, Chronos) for 952 cancer cell lines were obtained from the DepMap portal (https://depmap.org/portal/).

### Conditioned media assays

UM-Chor1-Cas9 cells were seeded at a density of 300,000 cells/well in six-well collagen I-coated plates. Cells were transduced with lentivirus as described in the Methods section corresponding to CRISPR-Cas9 screening validation. Following selection for infected cells with media containing 2 µg/mL puromycin, selective growth media was replaced with standard growth media. Three days post-media-change, conditioned media was harvested from cells and centrifuged to remove any residual cells. The supernatant was used to replace the standard growth media of UM-Chor1 cells previously seeded in 24-well plates (seeding density of 30,000 cells/well), according to the following three experimental conditions performed in parallel: supernatant alone, supernatant treated with neutralizing antibodies specific to type I IFNs (PBL Assay Science human type 1 IFN neutralizing antibody mixture, #39000; 1:50 dilution), or supernatant treated with neutralizing antibodies specific to IFN-β (PBL Assay Science anti-human IFN-beta, #31401; 1:100 dilution). Four replicate wells were treated per supernatant condition. Cells were counted after 5 days of treatment with conditioned media. Cell counts were obtained with a Vi-CELL XR Cell Viability Analyzer (Beckman Coulter), using Vi-CELL XR software (version 2.04; Beckman Coulter). Statistical analyses were performed with GraphPad Prism 9. The experiment was performed three times, with minor modifications to infection selection conditions.

### In vivo xenograft studies

Animal experiments were performed at XenoSTART in San Antonio, TX in tumor-bearing mice under an institutional animal care and use committee (IACUC)-approved protocol (#09-001, #10-001). Housing conditions for the mice included a room temperature of 70–74 °F, 30–60% relative humidity, and 12-h light/dark cycles. Six- to twelve-week-old female athymic nude mice (Charles River Laboratories) were implanted subcutaneously with low-passage tumor fragments. When tumors reached ~150–300 mm^3^ (for efficacy studies) or ~300–500 mm^3^ (for the pharmacodynamics study), animals matched by tumor volume (TV) were randomly allocated into control and treatment groups, with each group containing four to seven animals. RMC-4550 was formulated in 2% hydroxypropyl methylcellulose E-50, 0.5% Tween-80 in 50 mM sodium citrate buffer, pH 4.0. TNO155 was formulated in 0.5% Tween-80, and 0.5% methylcellulose. Dosing began at day 0 and drugs (or respective vehicles) were administered orally at the dose levels and schedules noted in the main text. Animals were observed daily, and weights and TVs were measured twice a week using an electronic scale and digital calipers, respectively. Tumor dimensions were converted to TV using the formula: TV (mm^3^) = width^2^ (mm) × length (mm) × 0.52. The study endpoints were when the control group reached mean TV = 1500 mm^3^ or a specified time point. Any individual animal reaching a tumor size >2.5 cm^3^ was subsequently removed from the study, in accordance with the XenoSTART IACUC protocol. For mice that reached the tumor volume endpoint before the study endpoint (one vehicle-treated mouse on day 25 of the CF539/TNO155 study), the final TV measurement was carried over and plotted for the remainder of the study, and body weight measurements were not plotted for this mouse beyond this date. Statistical analyses were performed with GraphPad Prism 9.

Immunoblotting of tumor tissue was performed by first homogenizing tumor fragments in lysis buffer (recipe described in the Immunoblotting methods section) using the Precellys Evolution instrument (Bertin Technologies) and the following protocol: 3 × 7500 rpm for 20 s, pausing for 15 s between rounds. Homogenized lysates were subsequently centrifuged for 2 min at 15,700 × *g*. Supernatants were quantified and subjected to immunoblotting as described in the Immunoblotting methods section. Immunoblots were performed twice.

### Immunohistochemical staining for Ki67 or cleaved caspase-3 expression

Terminal tumors were collected following the final dose and immediately formalin-fixed and then embedded in paraffin blocks. Histology was performed by HistoWiz Inc. (http://www.histowiz.com/). Paraffin-embedded samples were sectioned at 4 μm, and immunohistochemistry was performed on a Bond Rx autostainer (Leica Biosystems) with heat-mediated antigen retrieval using Epitope Retrieval Solution 1 (Leica Biosystems) for 20 min. Samples were treated with rabbit polyclonal Ki67 primary antibody (Abcam ab15580, 1:800 dilution) or cleaved caspase-3 primary antibody (Cell Signaling Technology #9661, 1:300 dilution), followed by an anti-rabbit HRP conjugated polymer system. Bond Polymer Refine Detection (Leica Biosystems) was used according to the manufacturer’s protocol. After staining, sections were dehydrated and film coverslipped using a TissueTek-Prisma and Coverslipper (Sakura). Whole slide scanning (40x) was performed on an Aperio AT2 (Leica Biosystems). Immunohistochemistry was performed once.

Image analysis was carried out using Halo image analysis software (version 3.4.2986.231; Indica Labs). Positive and negative cells were identified using the Halo Multiplex IHC algorithm (version 3.4.1) by first defining the settings for the hematoxylin counterstain to detect all nuclei, followed by setting thresholds to detect Ki67 or cleaved caspase-3 staining at weak, moderate, and strong intensities (Halo threshold settings of 0.30, 0.45, 0.60, respectively for Ki67; and 0.22, 0.40, 0.55, respectively for cleaved caspase-3). The percentage of Ki67- or cleaved caspase-3-positive cells reflects cumulative positive staining (weak, moderate, or strong intensity). The H-score was calculated as a weighted sum of the percentage of positive cells at each intensity, using the following convention: weak positive (× 1) + moderate positive (×2) + strong positive (×3), giving a possible range of 0 to 300. Statistical analyses were performed with GraphPad Prism 9.

### Statistics and reproducibility

Sample sizes were determined on the basis of published findings^17^ that had used similar sample sizes to detect a significant difference between groups. The reproducibility of the experimental findings was verified by performing some combination of the following: confirming an effect across different cell lines or tumor samples, replicating findings with independent experiments, using biological and technical replicates, using multiple concentrations of a compound, using multiple sgRNAs targeting a gene of interest, and confirming ex vivo results in different mouse model systems. The number of times each experiment was performed varied by experiment (ranging from one to five times) and is stated in the relevant Methods section. For the in vivo study using the CF539 model, one mouse in the vehicle group died of an unknown cause before the study endpoint and was therefore excluded from the analysis and figure. For the in vivo study using the U-CH1 model and TNO155 treatment, any data collected after the pre-established study endpoint were excluded from the analysis and figure. Individual microtiter wells not meeting routine quality control standards for proper cell seeding (pertains to some DMSO-treated wells using U-CH1 cells in the experiment depicted in Fig. 5b) were excluded from the analysis and figure. For in vivo studies, when tumors reached ~150–300 mm^3^ (for efficacy studies) or ~300–500 mm^3^ (for the pharmacodynamics study), animals matched by tumor volume were randomly allocated into control and treatment groups. Other studies were performed using a single population of cells that was randomly allocated into experimental groups before treatment. The investigators were not blinded to allocation during experiments and outcome assessment.

### Reporting summary

Further information on research design is available in the Nature Portfolio Reporting Summary linked to this article.

## Supplementary information

Supplementary Information

Reporting Summary

Description of Additional Supplementary Files

Supplementary Data 1

Supplementary Data 2

Supplementary Data 3

Supplementary Data 4

Supplementary Data 5

Supplementary Data 6

Supplementary Data 7

## Data Availability

CRISPR-Cas9 screening data for chordoma and non-chordoma cell lines are available through the Broad Institute Cancer Dependency Map 20Q2 release (https://depmap.org/portal/download/; 10.6084/m9.figshare.12280541.v4). Gene dependency and selectivity scores for chordoma cell lines (pertains to Fig. 1b and Supplementary Figs. 2, 15) are provided in Supplementary Data 1, 2. Genomic and transcriptomic correlates of selected dependency genes (pertains to Fig. 3) are available at Figshare (10.6084/m9.figshare.21774746.v1). GSEA results (pertains to Supplementary Fig. 8a) are provided in Supplementary Data 4. RNA-sequencing data generated from sgRNA-treated cells (pertains to Fig. 4b) are publicly available at Gene Expression Omnibus (GEO) under accession code GSE226734 (https://www.ncbi.nlm.nih.gov/geo/query/acc.cgi?acc=GSE226734) and Supplementary Data 5. The hg38 reference genome sequence and annotation are publicly available from the UCSC (http://genome.ucsc.edu/cgi-bin/hgGateway?db=hg38) or Ensembl (https://useast.ensembl.org/Homo_sapiens/Info/Index) genome browsers. AUC and EC_50_ values resulting from small-molecule sensitivity analysis (pertains to Fig. 5b) are provided in Supplementary Data 6. Source data are provided with this paper.

## Source data

Source Data
