# Supplementary material for: Mapping the landscape of genetic dependencies in chordoma
Source: Nat Commun. 2023 Apr 6;14:1933. doi: 10.1038/s41467-023-37593-8 (PMC10079670; doi:10.1038/s41467-023-37593-8)
Supplement: Supplementary file 1 — Supplementary Information [file 41467_2023_37593_MOESM1_ESM.pdf]

SUPPLEMENTARY INFORMATION

Mapping the landscape of genetic dependencies in chordoma

Sharifnia, Wawer et al.

Contents

Supplementary Figures 1-15

Supplementary Tables 1-2

Supplementary Information References

Uncropped immunoblots associated with Supplementary Figure 4

Uncropped immunoblots associated with Supplementary Figure 11

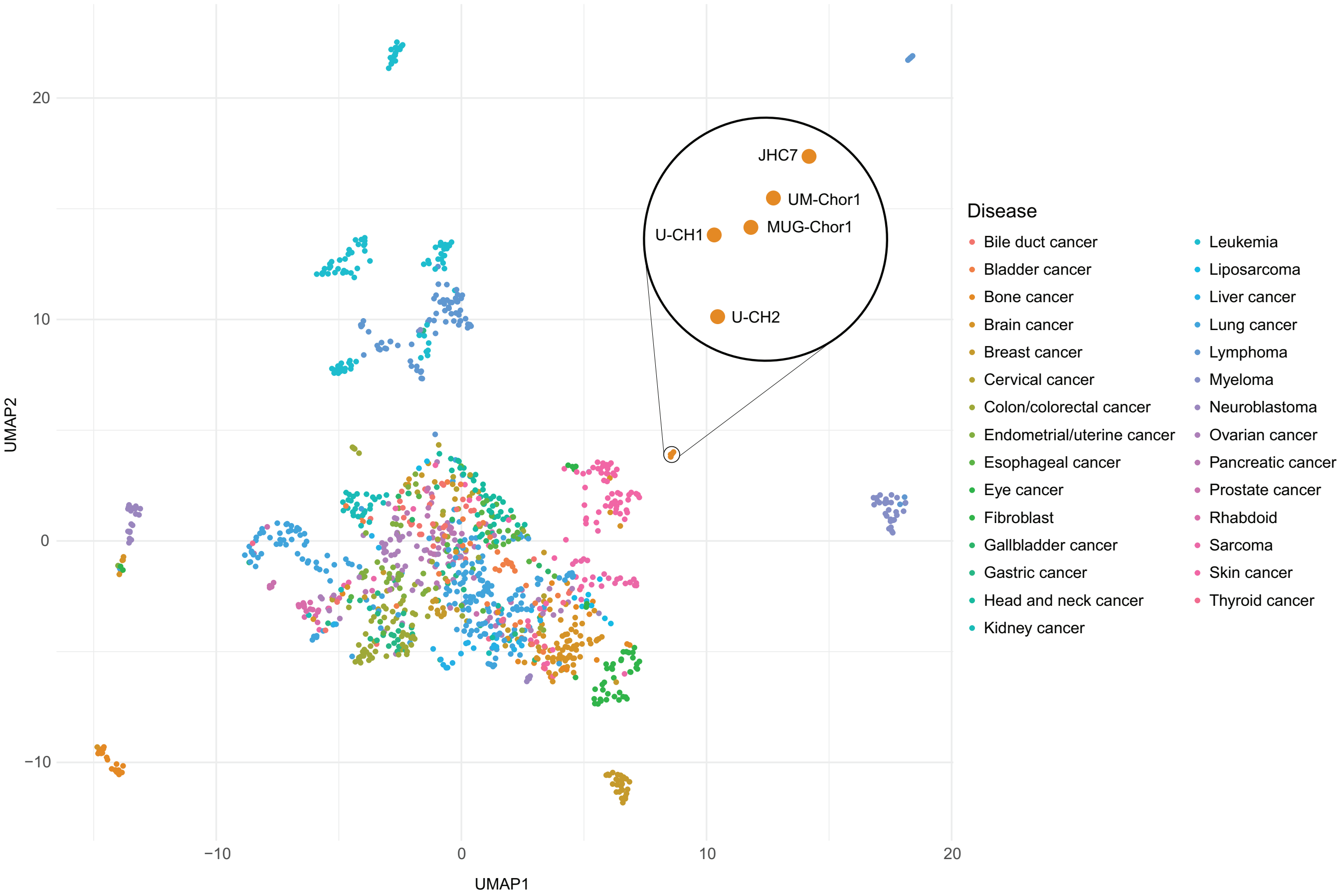

Supplementary Fig. 1. Similarity of gene-expression profiles of chordoma cell lines used for genome-scale CRISPR-Cas9 screens. Uniform manifold approximation and projection (UMAP) plot showing that chordoma cell lines form a distinct cluster based on gene-expression profiles generated from chordoma cell lines (including the U-CH1 chordoma cell line, which was not subjected to CRISPR-Cas9 screening) and 1,294 non-chordoma cancer cell lines in the CCLE, colored by lineage annotation. Source data are provided as a Source Data file.

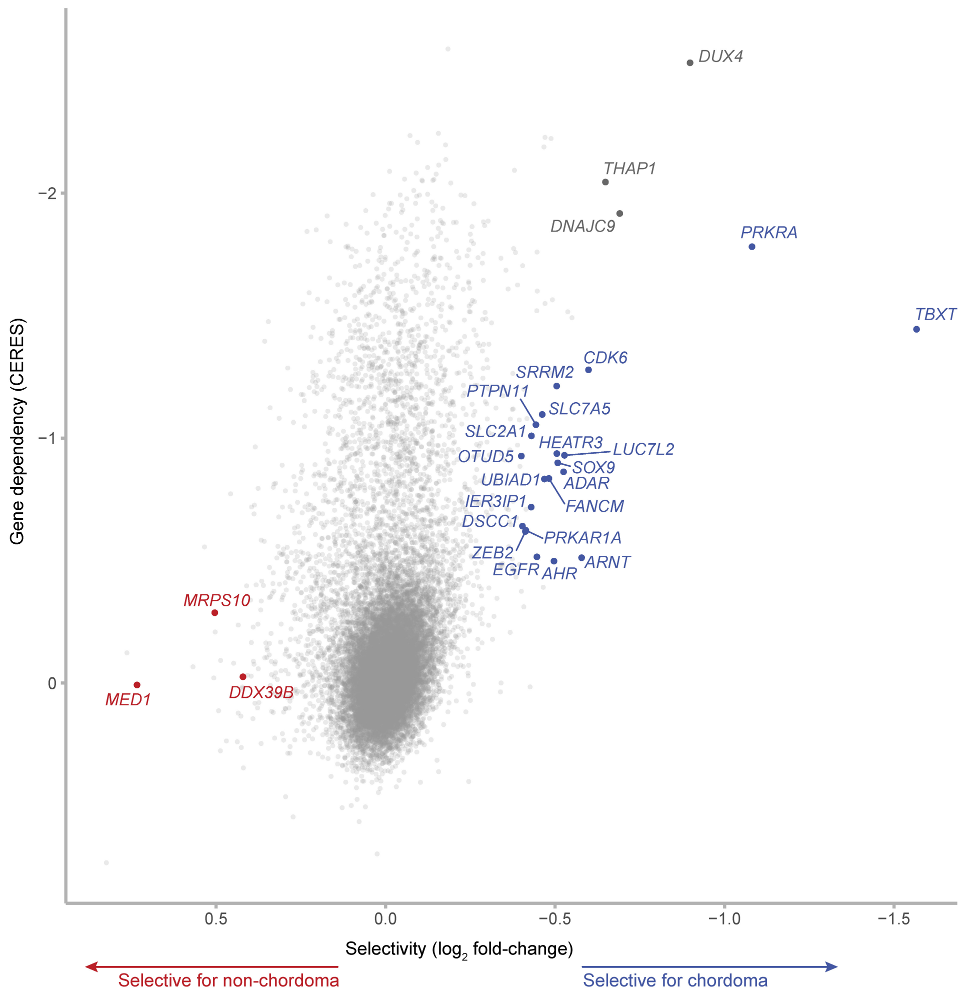

Supplementary Fig. 2. Genome-scale CRISPR-Cas9 screens identify a spectrum of selectively essential genes in chordoma (CERES analysis). Selective essentiality analysis identifying chordoma dependency genes. Selectivity is quantified by the log_2 fold-change in mean CERES gene effect scores between four chordoma and 765 non-chordoma cell lines (x-axis). The y-axis depicts the median CERES gene effect score for the chordoma cell lines. Lower CERES gene effect scores indicate higher dependency of a cell line on a given gene. Gene dependencies selective for chordoma/non-chordoma are indicated in blue/red (see Methods for details). Genes that do not meet the selectivity threshold for dependency probability scores (log_2 fold-change > 0.3) but are strongly selective for chordoma based on CERES gene effect scores only (log_2 fold-change > 0.5) are indicated and labeled in dark gray. They represent commonly essential genes that show a higher degree of viability effects in chordoma cell lines compared to non-chordoma cell lines. See also related Supplementary Data 2.

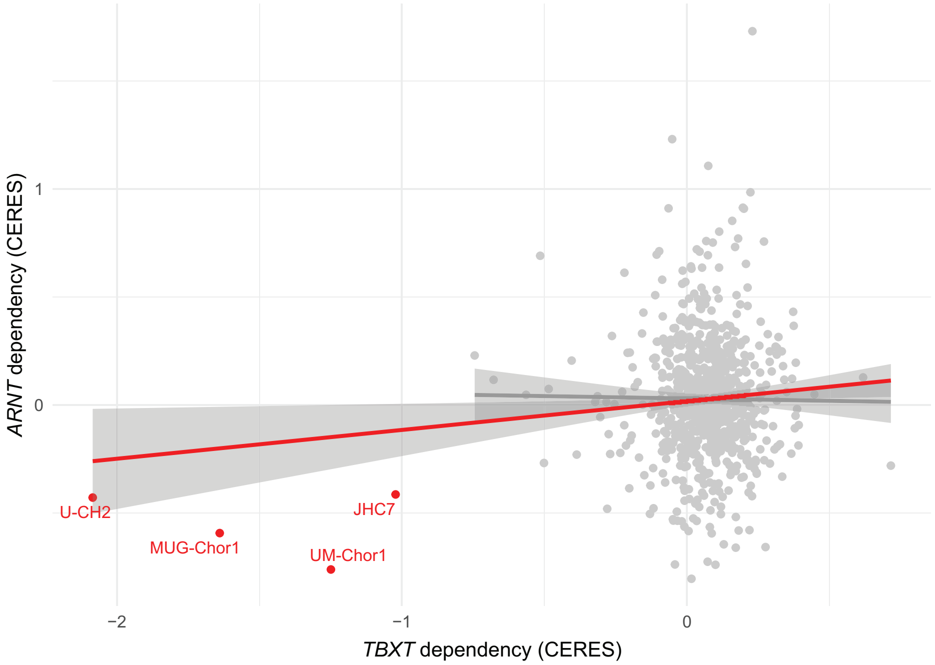

Supplementary Fig. 3. The correlation between *TBXT* and *ARNT* is driven by chordoma cell lines. Scatter plot of *TBXT* vs. *ARNT* CERES gene effect scores for all cell lines annotated with both *TBXT* and *ARNT* dependency (n = 769). Linear regression models indicate that the relationship between *TBXT* and *ARNT* is significant only when chordoma cell lines are included (red line, y = 0.017 + 0.133x, P = 0.020, derived from a two-tailed, one-sample t test for the slope coefficient). Without chordoma cell lines, the association is not significant (gray line, y = 0.030 – 0.022x, P = 0.772, derived from a two-tailed, one-sample t test for the slope coefficient). Gray areas around the regression lines indicate the 95% confidence intervals for each fit. Source data are provided as a Source Data file.

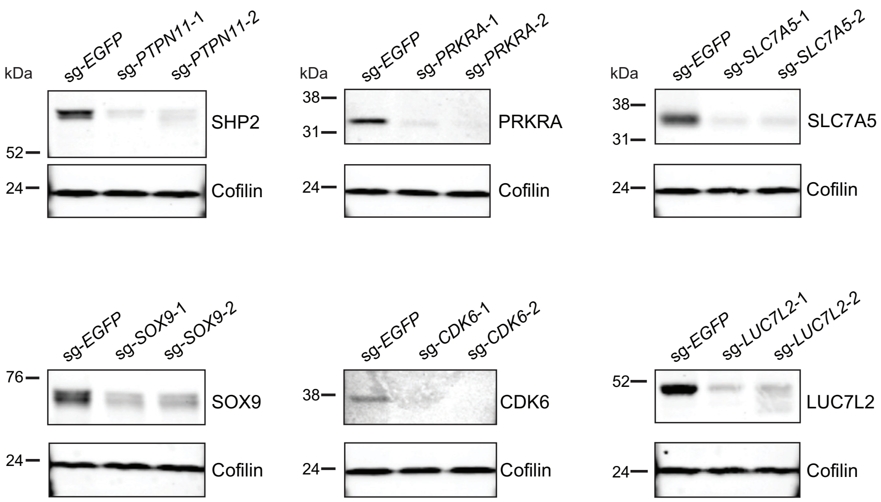

Supplementary Fig. 4. Immunoblot analysis confirming sgRNA-mediated protein repression. Immunoblot analysis of Cas9-expressing UM-Chor1 chordoma cells transduced with sgRNAs targeting a candidate dependency gene or a non-targeting sgRNA control.

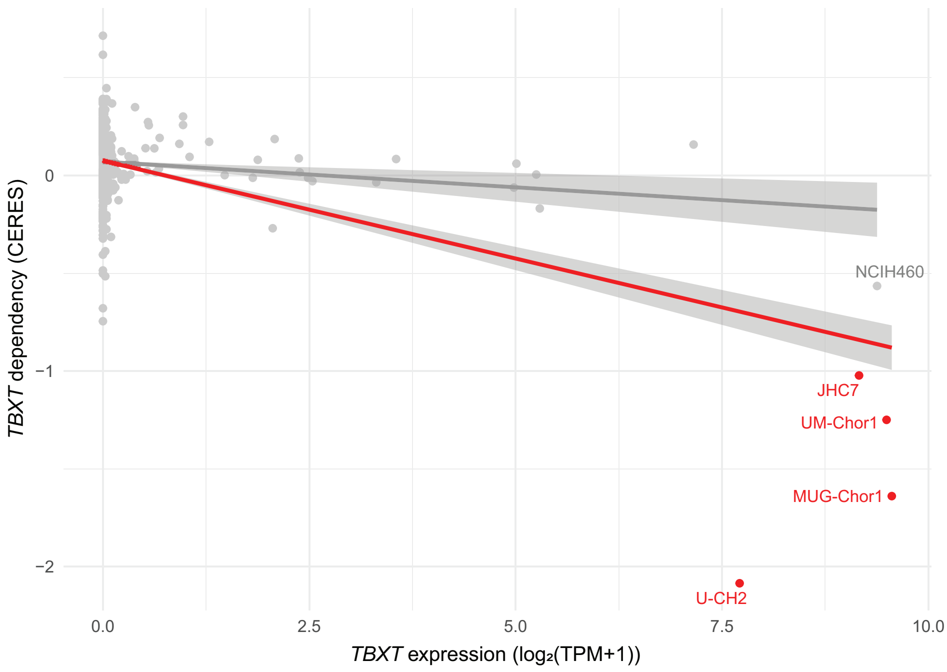

Supplementary Fig. 5. The correlation between *TBXT* dependency and *TBXT* expression is driven by chordoma cell lines. Scatter plot of *TBXT* gene expression vs. CERES gene effect scores for all cell lines annotated with both *TBXT* expression and dependency (n = 767). Linear regression models indicate that the relationship between *TBXT* dependency and *TBXT* expression is highly significant when chordoma cell lines are included (red line, y = 0.075 – 0.100x, $P = 2.22\times10^{-51}$, derived from a two-tailed, one-sample t test for the slope coefficient). Without chordoma cell lines, the association is much weaker (gray line, y = 0.071 – 0.026x, $P = 6\times10^{-4}$, derived from a two-tailed, one-sample t test for the slope coefficient). Additionally omitting NCIH460 (H460), a lung cancer cell line with known *TBXT* expression and dependency[1], leads to a non-significant association (line not shown, y = 0.070 – 0.010x, P = 0.255, derived from a two-tailed, one-sample t test for the slope coefficient). Gray areas around the regression lines indicate the 95% confidence intervals for each fit. Source data are provided as a Source Data file.

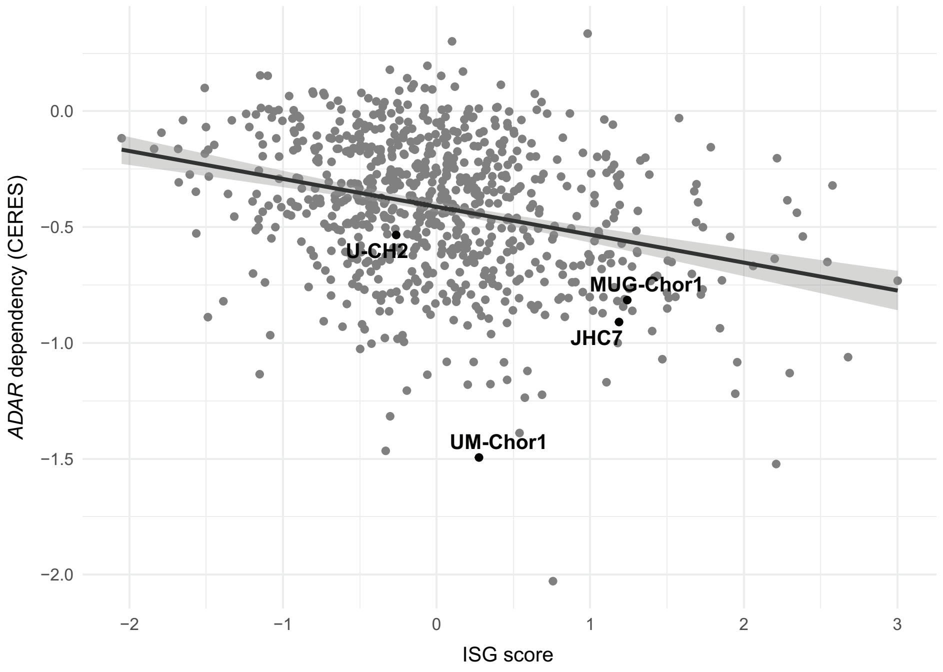

Supplementary Fig. 6. The ISG score is a predictor of *ADAR* dependency. Scatter plot of ISG scores vs. *ADAR* CERES gene effect scores for n = 767 cancer cell lines, overlayed with a regression line (y = –0.413 – 0.120x, P = 9.37×10^{-17}, derived from a two-tailed, one-sample t test for the slope coefficient). Chordoma cell lines are indicated. Gray areas around the regression lines indicate the 95% confidence intervals for each fit. Source data are provided as a Source Data file.

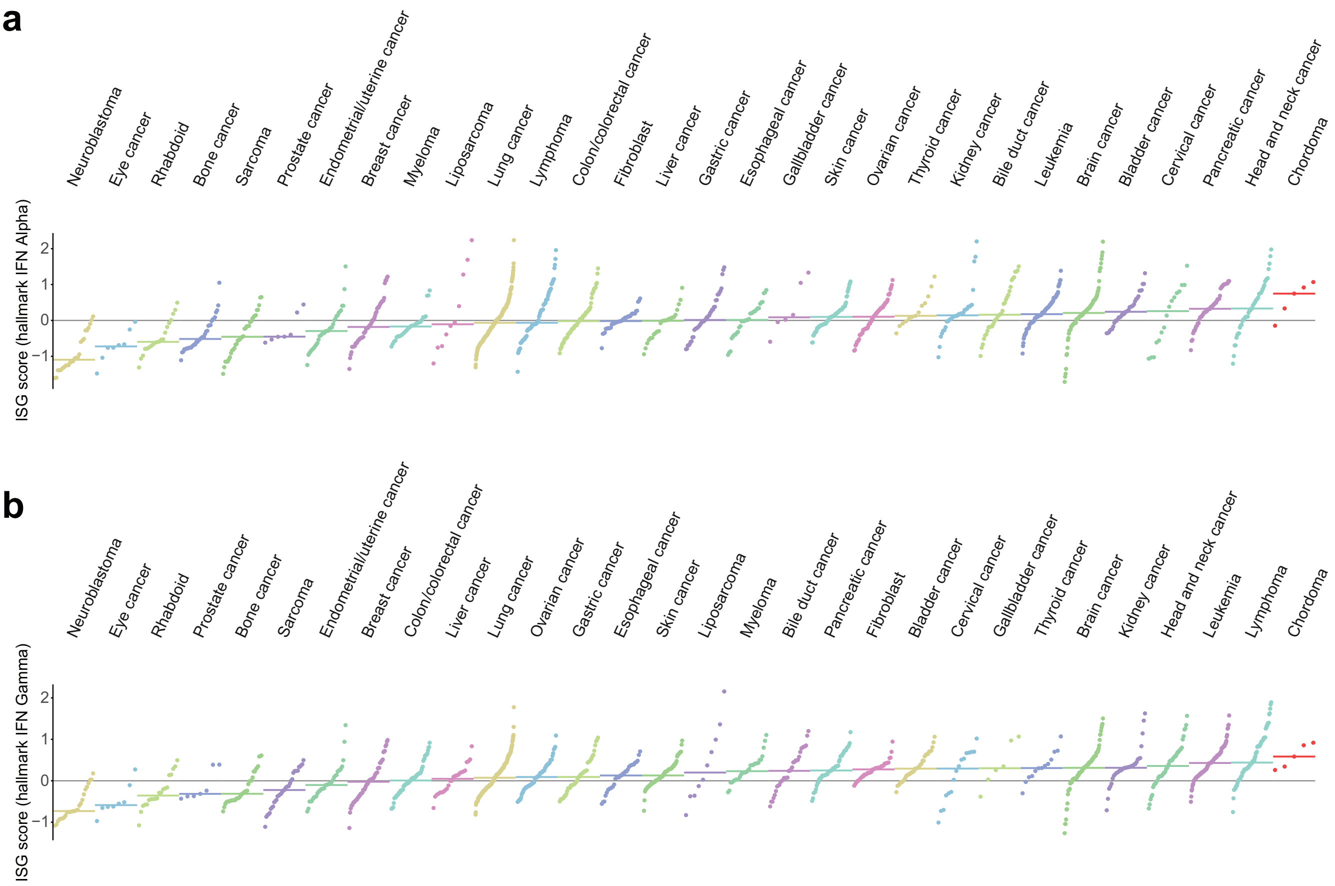

Supplementary Fig. 7. Alternative gene-set signatures confirm the high levels of interferon-stimulated gene expression in chordoma cell lines. Plots analogous to the ISG core score calculations depicted in Fig. 4a, using alternative interferon-related gene signatures from MSigDB[2] in place of the 38-gene signature[3]. **a** Distribution of ISG scores calculated with the MSigDB hallmark Interferon Alpha signature for chordoma cell lines and 1,294 non-chordoma cancer cell lines in the CCLE, grouped by lineage annotation. **b** Distribution of ISG scores calculated with the MSigDB hallmark Interferon Gamma signature for chordoma cell lines and 1,294 non-chordoma cancer cell lines in the CCLE, grouped by lineage annotation. Colored horizontal bars: median scores for each group. Gray horizontal line: zero-score mark. Source data are provided as a Source Data file.

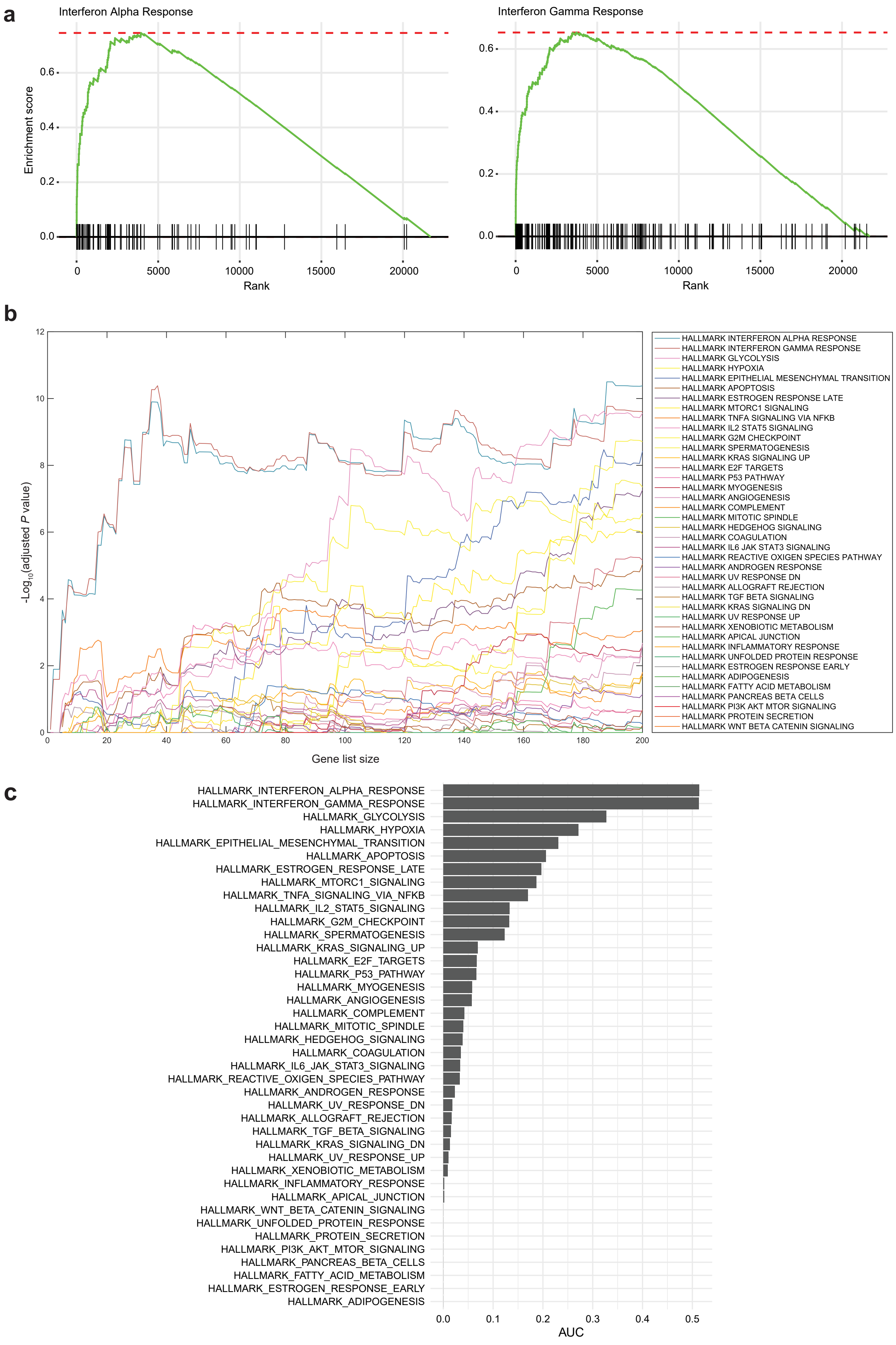
a
Interferon Alpha Response
Interferon Gamma Response
Enrichment score
0.0
0.2
0.4
0.6
0
5000
10000
15000
20000
Rank
b
-Log10(adjusted P value)
Gene list size
0
20
40
60
80
100
120
140
160
180
200
2
4
6
8
10
12
HALLMARK INTERFERON ALPHA RESPONSE
HALLMARK INTERFERON GAMMA RESPONSE
HALLMARK GLYCOLYSIS
HALLMARK HYPOXIA
HALLMARK EPITHELIAL MESENCHYMAL TRANSITION
HALLMARK APOPTOSIS
HALLMARK ESTROGEN RESPONSE LATE
HALLMARK MTORC1 SIGNALING
HALLMARK TNFA SIGNALING VIA NFKB
HALLMARK IL2 STAT5 SIGNALING
HALLMARK G2M CHECKPOINT
HALLMARK SPERMATOGENESIS
HALLMARK KRAS SIGNALING UP
HALLMARK E2F TARGETS
HALLMARK P53 PATHWAY
HALLMARK MYOGENESIS
HALLMARK ANGIOGENESIS
HALLMARK COMPLEMENT
HALLMARK MITOTIC SPINDLE
HALLMARK HEDGEHOG SIGNALING
HALLMARK COAGULATION
HALLMARK IL6 JAK STAT3 SIGNALING
HALLMARK REACTIVE OXIGEN SPECIES PATHWAY
HALLMARK ANDROGEN RESPONSE
HALLMARK UV RESPONSE DN
HALLMARK ALLOGRAFT REJECTION
HALLMARK TGF BETA SIGNALING
HALLMARK KRAS SIGNALING DN
HALLMARK UV RESPONSE UP
HALLMARK XENOBIOTIC METABOLISM
HALLMARK APICAL JUNCTION
HALLMARK INFLAMMATORY RESPONSE
HALLMARK UNFOLDED PROTEIN RESPONSE
HALLMARK ESTROGEN RESPONSE EARLY
HALLMARK ADIPOGENESIS
HALLMARK FATTY ACID METABOLISM
HALLMARK PANCREAS BETA CELLS
HALLMARK PI3K AKT MTOR SIGNALING
HALLMARK PROTEIN SECRETION
HALLMARK WNT BETA CATENIN SIGNALING
c
HALLMARK_INTERFERON_ALPHA_RESPONSE
HALLMARK_INTERFERON_GAMMA_RESPONSE
HALLMARK_GLYCOLYSIS
HALLMARK_HYPOXIA
HALLMARK_EPITHELIAL_MESENCHYMAL_TRANSITION
HALLMARK_APOPTOSIS
HALLMARK_ESTROGEN_RESPONSE_LATE
HALLMARK_MTORC1_SIGNALING
HALLMARK_TNFA_SIGNALING_VIA_NFKB
HALLMARK_IL2_STAT5_SIGNALING
HALLMARK_G2M_CHECKPOINT
HALLMARK_SPERMATOGENESIS
HALLMARK_KRAS_SIGNALING_UP
HALLMARK_E2F_TARGETS
HALLMARK_P53_PATHWAY
HALLMARK_MYOGENESIS
HALLMARK_ANGIOGENESIS
HALLMARK_COMPLEMENT
HALLMARK_MITOTIC_SPINDLE
HALLMARK_HEDGEHOG_SIGNALING
HALLMARK_COAGULATION
HALLMARK_IL6_JAK_STAT3_SIGNALING
HALLMARK_REACTIVE_OXIGEN_SPECIES_PATHWAY
HALLMARK_ANDROGEN_RESPONSE
HALLMARK_UV_RESPONSE_DN
HALLMARK_ALLOGRAFT_REJECTION
HALLMARK_TGF_BETA_SIGNALING
HALLMARK_KRAS_SIGNALING_DN
HALLMARK_UV_RESPONSE_UP
HALLMARK_XENOBIOTIC_METABOLISM
HALLMARK_INFLAMMATORY_RESPONSE
HALLMARK_APICAL_JUNCTION
HALLMARK_WNT_BETA_CATENIN_SIGNALING
HALLMARK_UNFOLDED_PROTEIN_RESPONSE
HALLMARK_PROTEIN_SECRETION
HALLMARK_PI3K_AKT_MTOR_SIGNALING
HALLMARK_PANCREAS_BETA_CELLS
HALLMARK_FATTY_ACID_METABOLISM
HALLMARK_ESTROGEN_RESPONSE_EARLY
HALLMARK_ADIPOGENESIS
0.0
0.1
0.2
0.3
0.4
0.5
AUC

Supplementary Fig. 8. Interferon alpha and interferon gamma response genes are significantly upregulated following *ADAR* gene suppression in chordoma. a Gene-set enrichment analysis (GSEA) plots for the two most-enriched gene sets from the hallmark collection, Interferon Alpha (94 genes, enrichment score = 0.75, normalized enrichment score = 2.34, Benjamini-Hochberg-adjusted P = 3.84×10^{-18}) and Interferon Gamma (188 genes, enrichment score = 0.65, normalized enrichment score = 2.16, Benjamini-Hochberg-adjusted P = 9.53×10^{-20}). **b** Benjamini-Hochberg-adjusted enrichment P values, derived from GeLiNEA's null model of degree-preserving random gene lists (see Methods), for all tested gene list sizes and gene sets. **c** Area-under-curve (AUC) values for GeLiNEA enrichment results. Source data are provided as a Source Data file. See also related Supplementary Data 4.

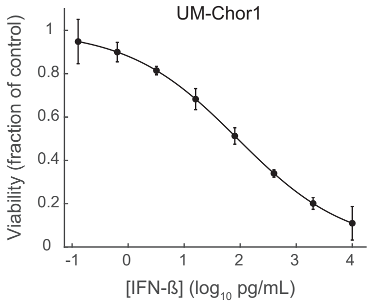

Supplementary Fig. 9. Chordoma cells are sensitive to IFN-β treatment. Viability of UM-Chor1 cells treated with indicated concentrations of IFN-β and assayed for cell viability after 6 d with CellTiter-Glo. Response data are represented by a fitted curve to the mean fractional viability at each concentration relative to vehicle-treated cells; error bars represent the s.e.m. ($n = 5$ biological samples measured in parallel). Source data are provided as a Source Data file.

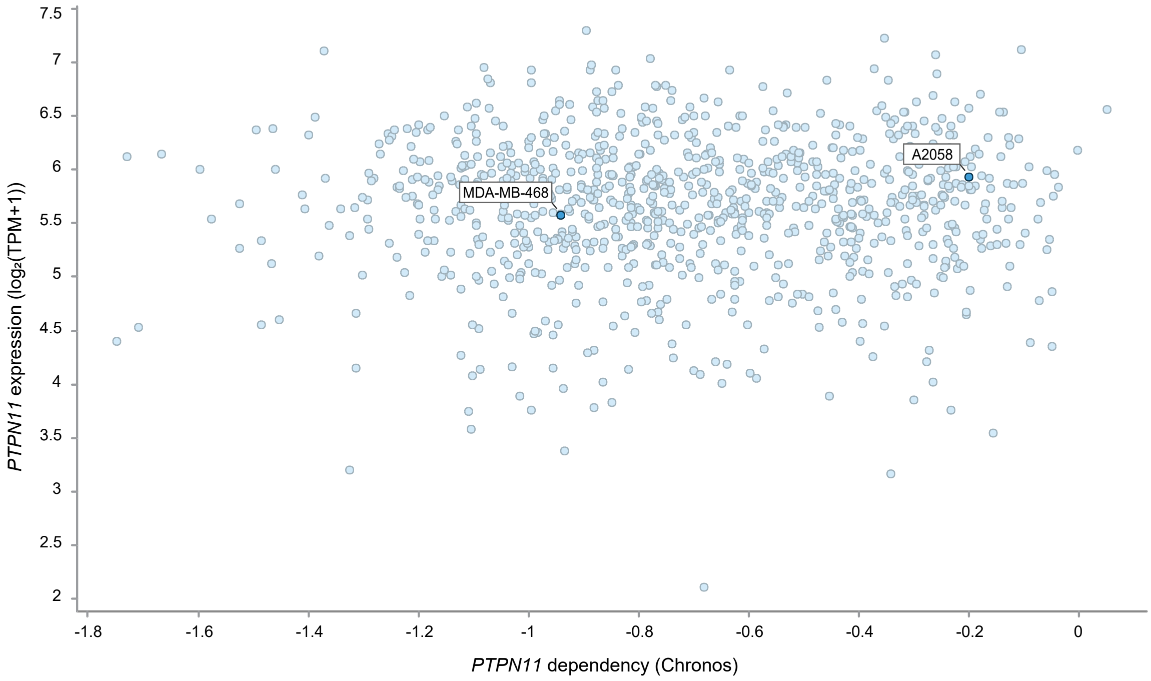

Supplementary Fig. 10. MDA-MB-468 and A2058 cell lines are sensitive and insensitive to loss of *PTPN11*, respectively. Data corresponding to *PTPN11* gene effect by CRISPR and *PTPN11* $\log_2$(TPM+1) expression for 952 cancer cell lines generated as part of the DepMap project. Gene effect is reported as a Chronos score[4]. Source data are provided as a Source Data file.

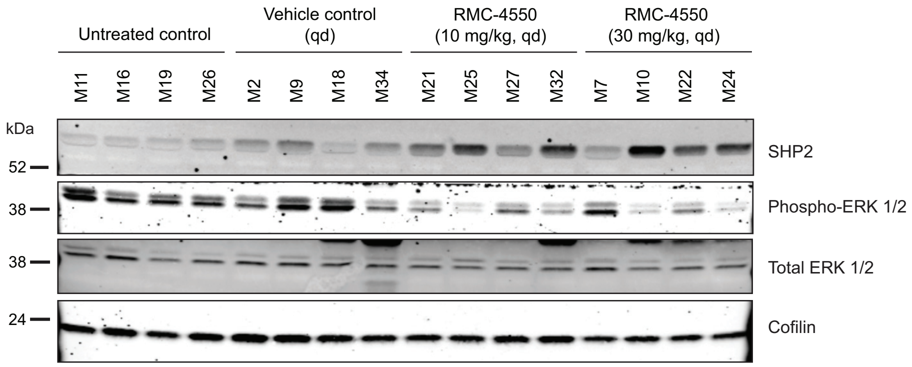

Supplementary Fig. 11. RMC-4550 treatment can reduce phosphorylation of ERK 1/2 *in vivo*. Immunoblot analysis of U-CH1 xenograft tumors following treatment with indicated doses of RMC-4550 once daily for 3 d.

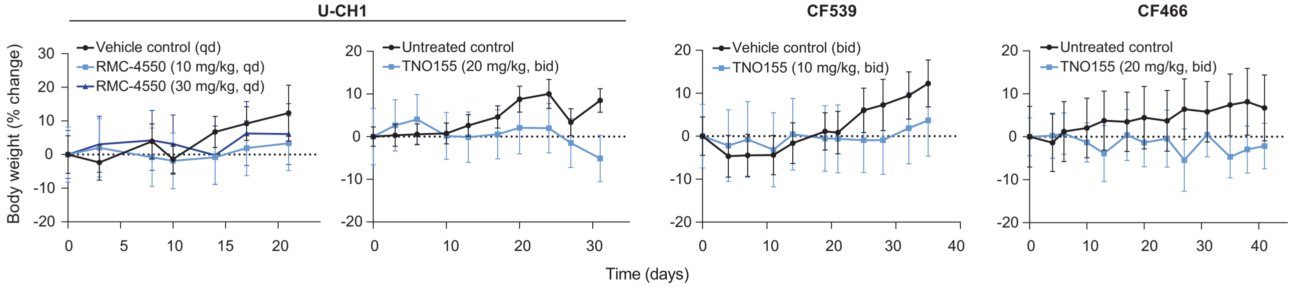

Supplementary Fig. 12. Tolerability of SHP2 inhibitor-treatment in mouse models of chordoma. Body weight (percent change relative to day 0 measurement) of mice engrafted with chordoma cells (U-CH1 cell line-derived xenograft, CF539 PDX, or CF466 PDX) and treated with a SHP2 inhibitor (RMC-4550 or TNO155). Points represent the mean body weight percent change ± s.e.m. ($n = 4$ (control) or 5 (compound) tumors for each arm of the U-CH1/RMC-4550 study; $n = 6$ (compound) or 7 (control) tumors for each arm of the U-CH1/TNO155 study; $n = 6$ (control) or 7 (compound) tumors for each arm of the CF539 study; $n = 7$ tumors for each arm of the CF466 study). Source data are provided as a Source Data file.

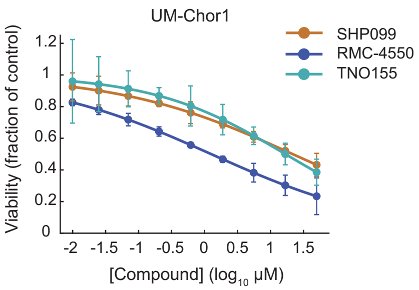

Supplementary Fig. 13. TNO155 has comparable potency to SHP099 and RMC-4550 in chordoma cells. Viability of UM-Chor1 cells treated with indicated concentrations of SHP2 inhibitors SHP099, RMC-4550, TNO155, or vehicle and assayed for cell viability after 6 d with CellTiter-Glo. Response data are represented by a fitted curve to the mean fractional viability at each concentration relative to vehicle-treated cells; error bars represent the s.e.m. (*n* = 4 biological samples measured in parallel). Source data are provided as a Source Data file.

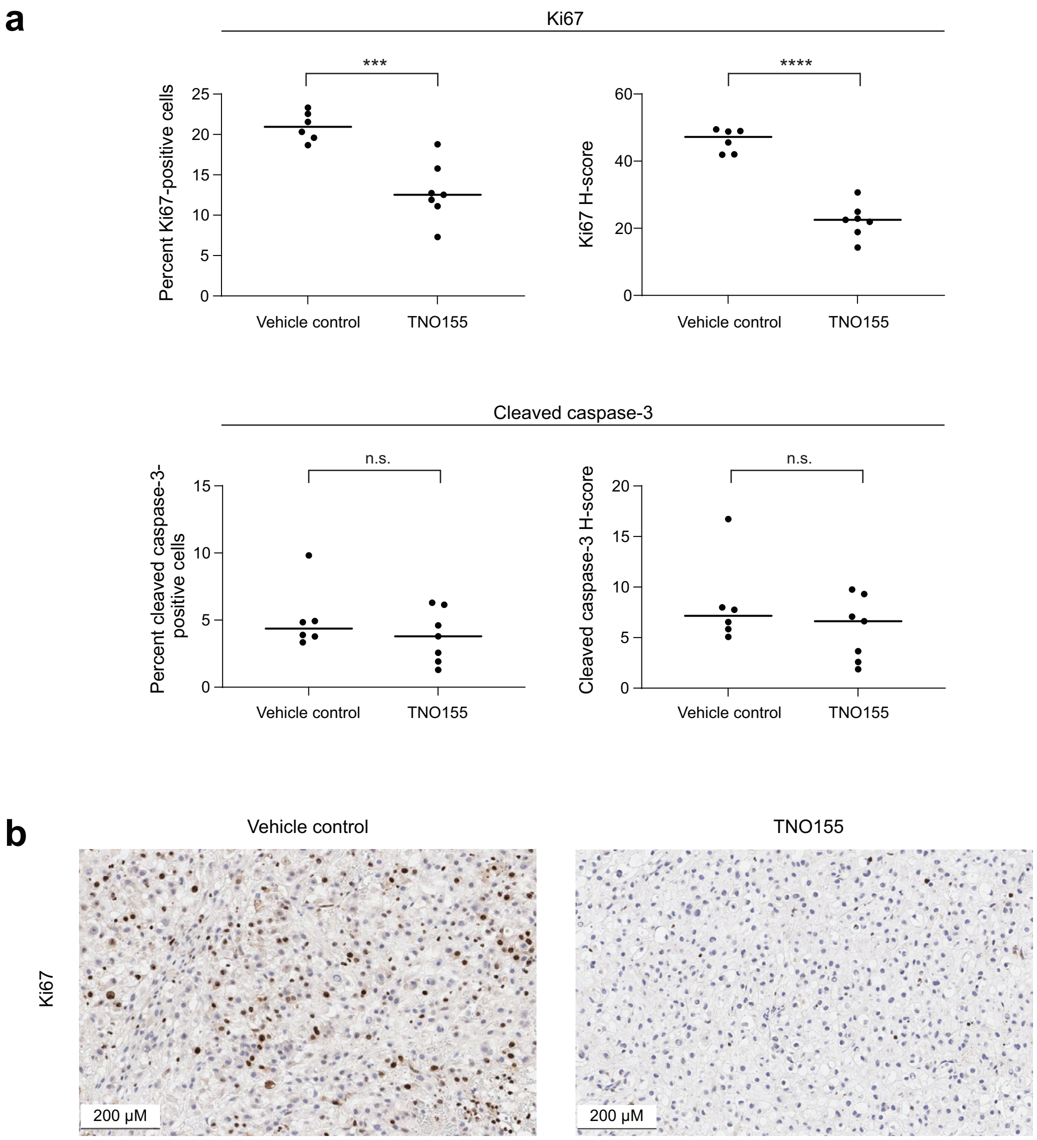

Supplementary Fig. 14. TNO155 treatment reduces tumor proliferation in the CF539 PDX model of chordoma. Immunohistochemical staining for Ki67 expression, a marker of cellular proliferation, or cleaved caspase-3 expression, a marker of apoptosis, using terminal tumor tissue from the CF539 experiment depicted in Fig. 5d (treatment with vehicle control bid or 10 mg/kg TNO155 bid). Tumor tissue was collected 2 h post-treatment for all mice, except for one vehicle-treated mouse whose tissue was collected 4 h post-treatment. **a** Quantification of Ki67- or cleaved caspase-3-positivity (top and bottom, respectively), by percentage of positive cells (left) or histochemical score (H-score; right), of tumor tissue collected from vehicle-treated (n = 6) and TNO155-treated (n = 7) mice and stained for Ki67 or cleaved caspase-3 expression. Horizontal lines indicate the median. n.s., not significant, ***P < 0.001, ****P < 0.0001, derived from a two-tailed, unpaired t test. Additional details of P values and effect sizes are reported in Supplementary Data 7. **b** Example images of tumor tissue collected from vehicle- or TNO155-treated mice and stained for Ki67 expression. Source data are provided as a Source Data file.

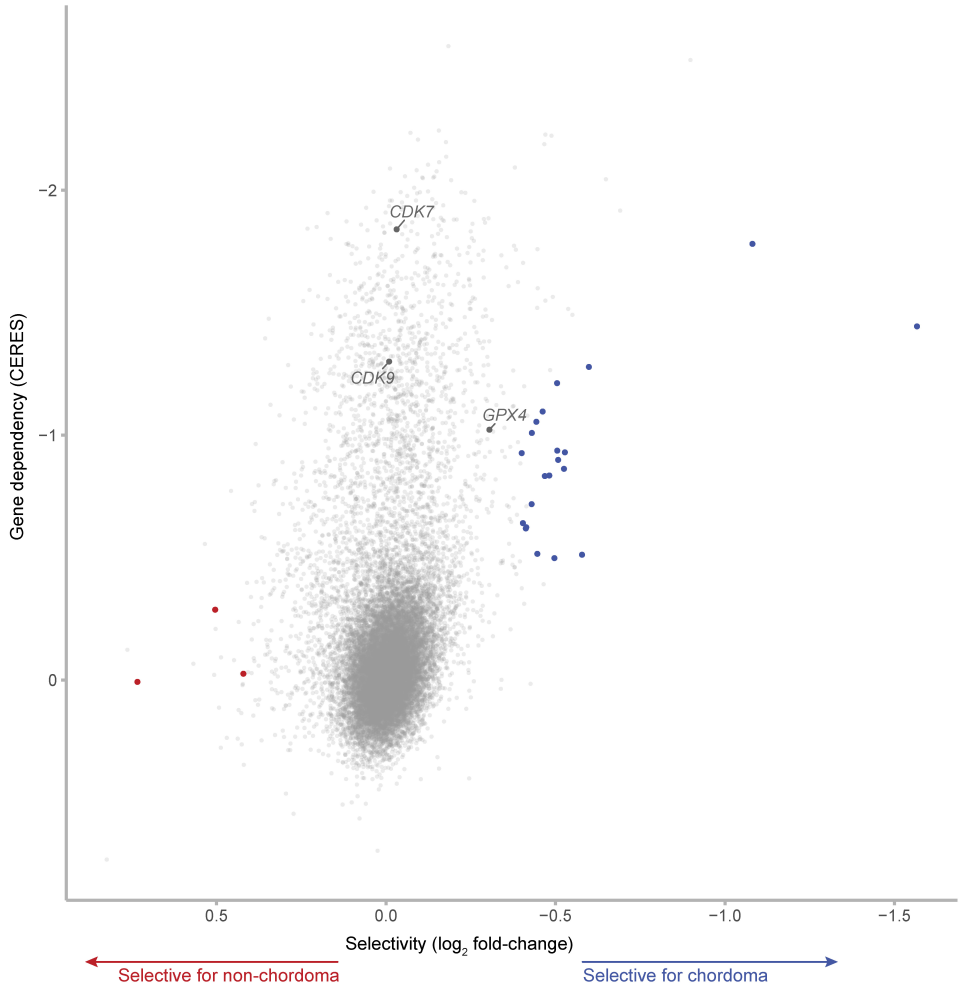

Supplementary Fig. 15. Genetic essentiality of known small-molecule dependencies in chordoma. Plot depicted in Supplementary Fig. 2, with positions of *CDK7*, *CDK9*, and *GPX4* indicated (dark gray). These genes encode proteins against which small-molecule inhibition was previously shown to have antiproliferative effects in chordoma models[5]. See also related Supplementary Data 2.

Cell line	Original location of disease	Disease status	Age
JHC7	Sacral	Primary	61
U-CH2	Sacral	Recurrent	72
MUG-Chor1	Sacral	Recurrent	57
UM-Chor1	Clival	Primary	66

Supplementary Table 1. Characteristics of chordoma cell lines used for genome-scale CRISPR-Cas9 screens. Chordoma cell lines used for genome-scale CRISPR-Cas9 screens, and the clinical characteristics associated with the originating tumors (source: www.chordomafoundation.org).

Location	Primer sequence (5' to 3')
sg-*ADAR*-1-F	AGAGGCCAGACCAGAACCAGCA
sg-*ADAR*-1-R	GCCCGCTGATGGGGTTCTTCAG
sg-*ADAR*-2-F	GGTCAGGAAGATTGGCGAGCTCG
sg-*ADAR*-2-R	TCCCTCAACTCGCCCCTTCTGT
sg-*CDK6*-1-F	AGGGCGCCTATGGGAAGGTGTT
sg-*CDK6*-1-R	TTCTGGGCCTGAGGATTCCCGG
sg-*CDK6*-2-F	CATGGAGAAGGACGGCCTGTGC
sg-*CDK6*-2-R	CACGTTGGGGTGCTCGAAGGTC
sg-*FANCM*-1-F	CCCTACCAATTGCCCAGTGCGG
sg-*FANCM*-1-R	TTTCGGCCATGTGGGATTGCGG
sg-*FANCM*-2-F	TGTGGCAAGATCATCCTTTGCCT
sg-*FANCM*-2-R	GGCAGAAAACTCATTCTTTACTGAGA
sg-*LUC7L2*-1-F	CCTTCCAGAGAATGGATCTTG
sg-*LUC7L2*-1-R	CTTACCCTTCATATAACCAGAGG
sg-*LUC7L2*-2-F	AGGGAATGTGGAGGAATCCCAGA
sg-*LUC7L2*-2-R	ACGCACCACCACAACCAGCTTT
sg-*PRKRA*-1-F	CGTCGGTGGCGGTTAAAACTGG
sg-*PRKRA*-1-R	TGAAGGTGAAAGTGGGCACGTGT
sg-*PRKRA*-2-F	TGGCATGCAAAGCACACCTTTT
sg-*PRKRA*-2-R	TGACTGCCAACCCACTCGGTCA
sg-*PTPN11*-1-F	AGCCTGAGCAAGGAGCGGGT
sg-*PTPN11*-1-R	GGCAGGAAATGAATGGGGAC
sg-*PTPN11*-2-F	CCCCTTGCCTCCCTTTCCAATGG
sg-*PTPN11*-2-R	GGCACAAGGGAGCAGCAGACTT
sg-*SLC2A1*-1-F	AACCTGCTCCCAGACACGCCTA
sg-*SLC2A1*-1-R	TCAGGTGGTGGCGTGAGACTGT
sg-*SLC2A1*-2-F	AAGGAAGACTGGGTCCTGGCCC
sg-*SLC2A1*-2-R	ACCGGCCAAAGCGGTTAACGAA
sg-*SLC7A5*-1-F	GGAGAAGATGCTGGCCGCCAAG
sg-*SLC7A5*-1-R	TTGGAGATGGTGGTGCCGAGCT
sg-*SLC7A5*-2-F	GAGAAGGAAGAGGCGCGGGAGA
sg-*SLC7A5*-2-R	CACGATGGAGAAGACGCCGCAC
sg-*SOX9*-1-F	GAACACGTTCCCCAAGGGCGAG
sg-*SOX9*-1-R	GTGCAAGTGCGGGTACTGGTCC
sg-*SOX9*-2-F	AGGAAGCCGAGTGGTCTGGGTC
sg-*SOX9*-2-R	TCTTCACCGACTTCCTCCGCCG
sg-*SRRM2*-1-F	ACCAAGACGAGGAAGATCCCGCA
sg-*SRRM2*-1-R	GCTGCGCCTCAAAGACAAGCGA
sg-*SRRM2*-2-F	CTCCCCACAACCCCTTGCAACC
sg-*SRRM2*-2-R	ACCCTGGAGCTGGAGCAGGTTT
sg-*THAP1*-1-F	CTTGGAGATGGGAGACGGGCGA
sg-*THAP1*-1-R	CGGGCTTGTCCTTGTCGTAGCG
sg-*THAP1*-2-F	GGCTGCAAGAACCGCTACGACA
sg-*THAP1*-2-R	TGTTCCAGGAGCGCGAGAAACG

Supplementary Table 2. Primer sequences associated with amplicon sequencing experiments.

Supplementary Information References

1 Xu, J. *et al.* The Role of Transcriptional Factor Brachyury on Cell Cycle Regulation in Non-small Cell Lung Cancer. *Front Oncol* **10**, 1078, doi:10.3389/fonc.2020.01078 (2020).

2 Liberzon, A. *et al.* The Molecular Signatures Database (MSigDB) hallmark gene set collection. *Cell Syst* **1**, 417-425, doi:10.1016/j.cels.2015.12.004 (2015).

3 Liu, H. *et al.* Tumor-derived IFN triggers chronic pathway agonism and sensitivity to ADAR loss. *Nat Med* **25**, 95-102, doi:10.1038/s41591-018-0302-5 (2019).

4 Dempster, J. M. *et al.* Chronos: a CRISPR cell population dynamics model. *bioRxiv*, 2021.2002.2025.432728, doi:10.1101/2021.02.25.432728 (2021).

5 Sharifnia, T. *et al.* Small-molecule targeting of brachyury transcription factor addiction in chordoma. *Nat Med* **25**, 292-300, doi:10.1038/s41591-018-0312-3 (2019).

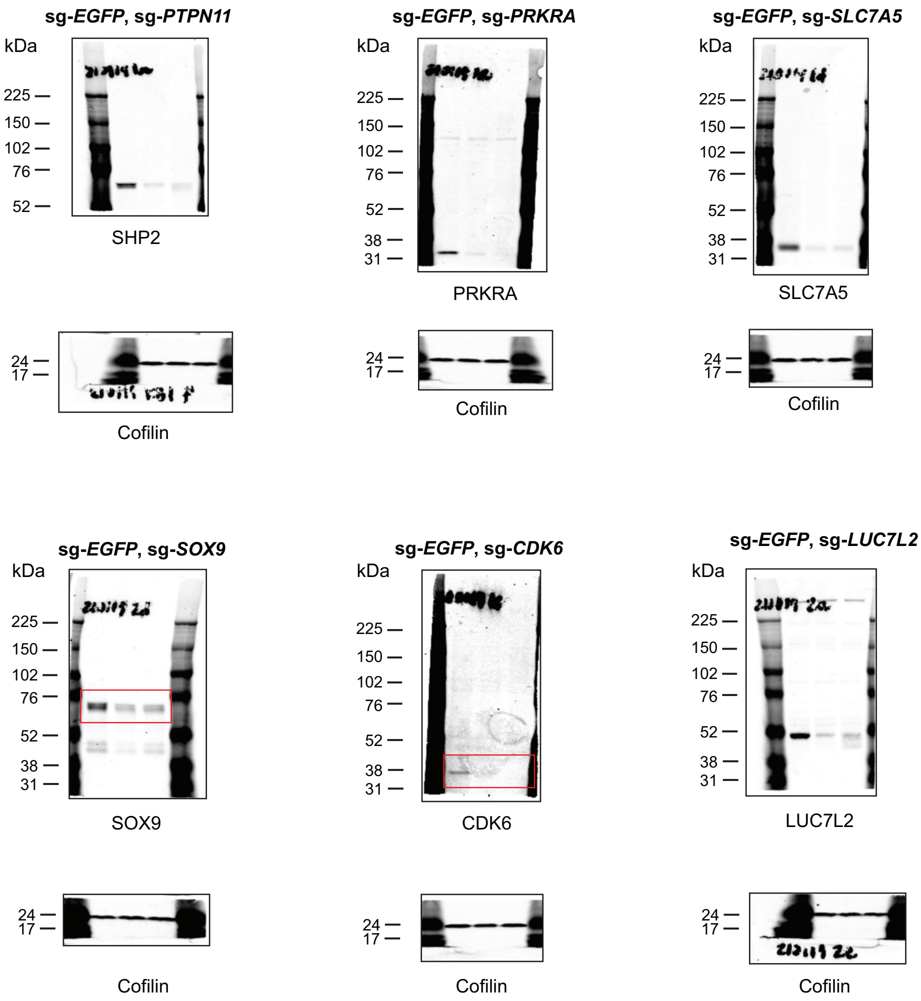

Uncropped immunoblots associated with Supplementary Fig. 4.

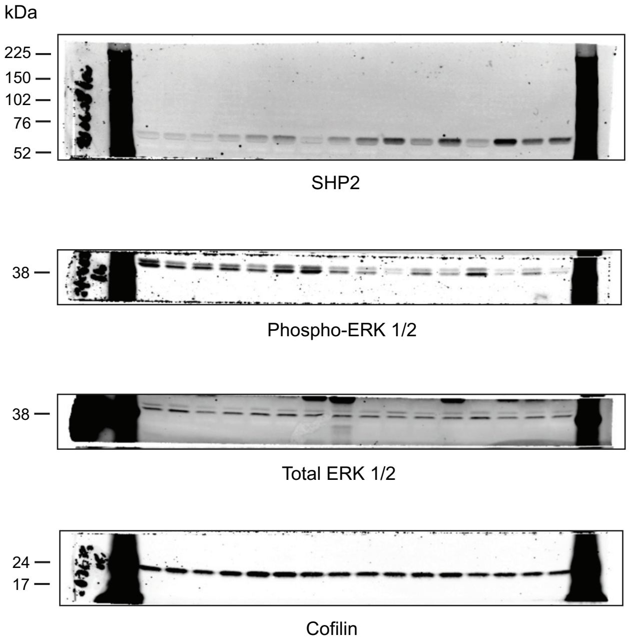

Uncropped immunoblots associated with Supplementary Fig. 11.
