# Supplementary material for: Mapping the landscape of genetic dependencies in chordoma
Source: Nat Commun. 2023 Apr 6;14:1933. doi: 10.1038/s41467-023-37593-8 (PMC10079670; doi:10.1038/s41467-023-37593-8)
Supplement: Supplementary file 3 — Description of Additional Supplementary Files [file 41467_2023_37593_MOESM3_ESM.docx]

**Description of Additional Supplementary Files**

**Supplementary Data 1. Gene dependency and selectivity scores (using probability scores) for chordoma cell lines following genome-scale CRISPR-Cas9 screening.** Genome-wide gene-dependency probability scores for 4 chordoma cell lines (U-CH2, UM-Chor1, MUG-Chor1, JHC7); median dependency probability scores across 4 chordoma cell lines and 765 non-chordoma cell lines; limma results determining the selectivity of gene dependencies (based on dependency probability scores) for 4 chordoma cell lines compared to 765 non-chordoma cell lines (log_2_ fold-change, *P* value derived from a two-tailed moderated *t* test, and Benjamini-Hochberg-adjusted *P* value; see Methods).

**Supplementary Data 2. Gene dependency and selectivity scores (using CERES scores) for chordoma cell lines following genome-scale CRISPR-Cas9 screening.** Genome-wide CERES gene-dependency scores for 4 chordoma cell lines (U-CH2, UM-Chor1, MUG-Chor1, JHC7); median CERES scores across 4 chordoma cell lines and 765 non-chordoma cell lines; limma results determining the selectivity of gene dependencies (based on CERES scores) for 4 chordoma cell lines compared to 765 non-chordoma cell lines (log_2_ fold-change, *P* value derived from a two-tailed moderated *t* test, and Benjamini-Hochberg-adjusted *P* value; see Methods).

**Supplementary Data 3. Status of selected genomic and transcriptomic features in chordoma cell lines.** (First tab) Copy number (log_2_(relative to ploidy + 1)) and z-scored copy number (based on mean and standard deviation per gene for 1742 cell lines available in the 20Q2 DepMap release, including chordoma cell lines) for copy-number correlates highlighted in Fig. 3 (*CDKN2A*, *CDKN2B*, *ADAR*, *SRRM2*, *LUC7L*) in four chordoma cell lines. (Second tab) Gene-expression values (log_2_(TPM+1)) and z-scored expression value (based on mean and standard deviation per gene for 1308 cell lines available in the 20Q2 DepMap release, including chordoma cell lines) for gene-expression correlates highlighted in Fig. 3 (*CDKN2A*, *CDKN2B*, *RB1*, *CDK6*, *ADAR*, *SRRM2*, *LUC7L*, *TBXT*, *PRKRA*) in four chordoma cell lines. No chordoma cell line had mutations in any of the mutation correlates highlighted in Fig. 3 (*RB1*, *BRAF*, *NRAS*, *KRAS*, *BRCA1*).

**Supplementary Data 4. Gene-set enrichment analysis (GSEA) results for gene-expression changes following *ADAR* gene suppression in UM-Chor1-Cas9 chordoma cells.** GSEA results for all gene sets in the MSigDB hallmark collection^1^. Each row corresponds to one gene set. pathway: name of the gene set; pval: enrichment *P* value derived from adaptive multi-level split Monte Carlo sampling; padj: Benjamini-Hochberg-adjusted *P* value; log2err: expected error for the standard deviation of the *P*-value logarithm; ES: enrichment score; NES: enrichment score normalized to mean enrichment of random samples of the same size; size: number of genes in the set. See documentation for the fgsea R package^2^ and the GSEA user guide (https://www.gsea-msigdb.org/gsea/doc/GSEAUserGuideTEXT.htm) for details.

**Supplementary Data 5. Differential gene-expression analysis following sgRNA-mediated *ADAR* suppression in chordoma cells.** Differential gene-expression results from DESeq2, comparing Cas9-expressing UM-Chor1 cells transduced with sgRNAs targeting *ADAR* (two replicates, each transduced with a distinct sgRNA) versus a non-targeting sgRNA control (*EGFP*, one replicate). Gene expression was measured with RNA sequencing. baseMean: mean of normalized counts for all samples; log2FoldChange: log_2_ fold-change in gene expression following sg-*ADAR* vs. sg-*EGFP* control treatment; lfcSE: standard error of log_2_ fold-change; stat: Wald test statistic; pvalue: Wald test *P* value; padj: Benjamini-Hochberg-adjusted *P* value.

**Supplementary Data 6.** **Concentration-response AUC and half-maximal effective concentration (EC_50_) values for small-molecule sensitivity experiments with RMC-4550 and SHP099.** cell_line: name of chordoma or control cell line; cpd_name: compound name; AUC: numerically integrated area under 3-parameter sigmoid fits of mean-fractional-viability concentration-response curves for each compound; EC50: effective concentration of half-maximal sensitivity, in µM, predicted by the curve fit (reported only if the prediction was within the concentration range tested); effect_size: expressed as percent inhibition (predicted lower asymptote of the curve fit).

**Supplementary Data 7. Statistical analyses and significance values.** Summary of the statistical tests performed, including *P* values and effect sizes, for the indicated figures.

**Supplementary Data References**

1 Liberzon, A. *et al.* The Molecular Signatures Database (MSigDB) hallmark gene set collection. *Cell Syst* **1**, 417-425, doi:10.1016/j.cels.2015.12.004 (2015).

2 Korotkevich, G. *et al.* Fast gene set enrichment analysis. *bioRxiv*, 060012, doi:10.1101/060012 (2021).
